# Large Differences in Gene Expression Responses to Drought and Heat Stress between Elite Barley Cultivar Scarlett and a Spanish Landrace

**DOI:** 10.3389/fpls.2017.00647

**Published:** 2017-05-01

**Authors:** Carlos P. Cantalapiedra, María J. García-Pereira, María P. Gracia, Ernesto Igartua, Ana M. Casas, Bruno Contreras-Moreira

**Affiliations:** ^1^Department of Genetics and Plant Production, Estación Experimental de Aula Dei (CSIC) Zaragoza, Spain; ^2^Fundación ARAID Zaragoza, Spain

**Keywords:** barley, landrace, drought, heat, transcriptome profiling, gene expression, RNAseq

## Abstract

Drought causes important losses in crop production every season. Improvement for drought tolerance could take advantage of the diversity held in germplasm collections, much of which has not been incorporated yet into modern breeding. Spanish landraces constitute a promising resource for barley breeding, as they were widely grown until last century and still show good yielding ability under stress. Here, we study the transcriptome expression landscape in two genotypes, an outstanding Spanish landrace-derived inbred line (SBCC073) and a modern cultivar (Scarlett). Gene expression of adult plants after prolonged stresses, either drought or drought combined with heat, was monitored. Transcriptome of mature leaves presented little changes under severe drought, whereas abundant gene expression changes were observed under combined mild drought and heat. Developing inflorescences of SBCC073 exhibited mostly unaltered gene expression, whereas numerous changes were found in the same tissues for Scarlett. Genotypic differences in physiological traits and gene expression patterns confirmed the different behavior of landrace SBCC073 and cultivar Scarlett under abiotic stress, suggesting that they responded to stress following different strategies. A comparison with related studies in barley, addressing gene expression responses to drought, revealed common biological processes, but moderate agreement regarding individual differentially expressed transcripts. Special emphasis was put in the search of co-expressed genes and underlying common regulatory motifs. Overall, 11 transcription factors were identified, and one of them matched *cis*-regulatory motifs discovered upstream of co-expressed genes involved in those responses.

## Introduction

Barley (*Hordeum vulgare* L.) is the fourth cereal crop in relevance worldwide. Like most crops, its production is affected by environmental stresses, drought being the most important among them (Cattivelli et al., 2008). Drought is already prominent at several major agricultural areas throughout the world (Luck et al., 2015), and its effects are predicted to worsen due to growing water demand, shrinking water supply and increased seasonal variability (Barnabas et al., 2008; Luck et al., 2015). An increment of overall temperature is also expected (Barnabas et al., 2008; Intergovernmental Panel on Climate Change, 2014). Actually, many stresses often occur in combination, as is the case of drought and heat, thus being more harmful (Challinor et al., 2014; Mickelbart et al., 2015). However, modern breeding has been directed mainly toward increasing yield, without considering yield stability as a major goal (Mittler, 2006). Therefore, attention is growing toward minimizing the gap between yields under optimal and stress conditions (Cattivelli et al., 2008), to cope with current yield variability (Keating et al., 2010), and to contribute to adaptation to global change (Challinor et al., 2014).

An appropriate strategy to achieve this goal is the exploitation of genetic diversity not yet incorporated into elite cultivars (Dwivedi et al., 2016). As in other crops, current barley cultivars exhibit a narrower genetic basis than wild progenitors (*Hordeum vulgare* ssp. *spontaneum*) and landraces, which are the primary source of useful genes for breeding programs (Fischbeck, 2003; Dawson et al., 2015). Furthermore, in environments with low productivity, landraces and old cultivars often outperform modern genotypes (Ceccarelli et al., 1998; Pswarayi et al., 2008; Yahiaoui et al., 2014). In comparison with wheat, barley has been grown in a wider range of environmental conditions, and is the predominant crop in marginal areas with little precipitation. Accordingly, it is sown in large expanses of the Mediterranean-climatic regions (Ceccarelli, 1994; Ryan et al., 2009), where drought can occur at any moment during the life cycle of crops, being particularly frequent during the terminal stages (Turner, 2004), when different components of grain yield can be largely influenced (Fischer and Turner, 1978; Saini and Westgate, 1999; Araus et al., 2002). Therefore, barley landraces adapted to such conditions could bear genes useful for breeding programs aiming to obtain better yields under drought.

Technical advances in the last decade have potential to improve crop breeding processes (Rivers et al., 2015). High throughput sequencing technologies are providing new powerful tools to study the association between plant genotypic and phenotypic variation (Varshney et al., 2014; Dawson et al., 2015). One of these, RNAseq (Mortazavi et al., 2008), is currently employed with different aims in crop genetics, like polymorphism detection and transcript profiling (Varshney et al., 2009). The latter can be used to analyze gene expression networks involved in different processes; for example, those related with resistance to abiotic stresses. However, analyses of *cis*-regulatory elements of transcription factors (TFs) and of promoters of genes involved in a given response have been rare in barley, likely due to the absence of adequate genomics resources.

In this work, two contrasting barley genotypes were subjected to prolonged water deficit, either alone or combined with heat. Spanish barley landrace SBCC073 was the best yielding genotype, among 159 landraces and 25 old and modern cultivars, in field trials in Spain in which average yield was below 3 t ha^−1^ (Yahiaoui et al., 2014). Also, it contributed QTLs for increased grain yield and early vigor in field experiments in Spain, particularly under drought, in a population in which SBCC073 was crossed with a highly productive cultivar, grown for three seasons with contrasting rainfall conditions (Boudiar et al., 2016). Therefore, SBCC073 is a landrace with valuable traits to be incorporated in breeding programs for improvement of barley for Mediterranean conditions. Here, it was compared to a modern cultivar, Scarlett, sensitive to water stress (Sayed et al., 2012). *De novo* assemblies of transcriptomes of both genotypes were obtained and gene expression changes evaluated both in developing inflorescences and leaves. Metabolic pathways, biological processes, molecular functions, co-expression clusters and *cis*-regulatory elements of drought-modulated genes are reported.

## Materials and methods

### Plant material and drought experiments

SBCC073 is a Spanish landrace-derived line, developed by three generations of head-row multiplication starting from a single seed of the original landrace, with no phenotypic selection. It is fully homozygous (http://www.eead.csic.es/EEAD/barley/core.php?var=73). Scarlett is a German spring malting cultivar. Seed was provided by Saatzucht Breun GmbH. SBCC073 is an intermediate growth habit type, according to Szucs et al. (2007), and it needs just 15 days of vernalization to promote timely flowering. Seeds of SBCC073 and Scarlett were sown and seedlings were allowed to grow for 1 week. Then, they were vernalized for 24 days, in order to synchronize the development of the two genotypes. At the end of the vernalization period, plants at the 3-leaf stage were transferred to 28.0 × 20.8 cm (height × diameter) black plastic pots (one seedling per pot) with standard substrate made of peat, fine sand and perlite Europerl B-10 (Europerlita Española SA, Barcelona, Spain), from a mix with 46 kg, 150 kg, and 50 L, respectively. Two series of pots were placed in a greenhouse (natural photoperiod, controlled maximum temperature 28°C, average daily temperature 25 ± 2°C during the day and 21 ± 3°C at night) and in a growth chamber (16 h light/8 h dark, 21°C daytime/18°C night temperature). Additional pots filled only with substrate were used to estimate dry weight and field capacity (FC). Soluble fertilizer was provided with irrigation. Plants were treated with fungicide (Triadimenol 25%) to prevent powdery mildew build-up.

Plants were subjected to a control and two different stress treatments: severe drought, and mild drought combined with heat. Control conditions and the severe drought treatment were imposed in a growth chamber. There, plants remained well-watered (70% FC) for 30 days. Then, water application was gradually reduced, to resemble a slow drying soil, based on weight of each pot relative to the estimated FC. Once the target fraction of FC was reached, the pots were watered to keep such weight constant. Treatment levels in the growth chamber were 70% (control) and 20% FC (severe drought). At the sampling date, 30 days after the beginning of the drought treatment, all plants had been at the target fraction of FC for at least 14 days. Plants in the greenhouse were subjected to heat stress right after transplant, for 61 days, from Zadoks growth stage (GS) 20 (Zadoks et al., 1974) up to GS 73, on average. Out of these, the first 30 days the plants were well-watered (80% FC). The last 31 days, the drought treatment was applied gradually, as in the growth chamber, targeting an intermediate drought stress level of 50% FC. During all 61 days, day temperature was kept under 30° with a cooling system, whereas temperature was not controlled at night. Average temperature over all period was 23.6°C (daily range 20.9°–26.3°C), average maximum temperature was 27.6°C, and average minimum temperature was 18.8°C. The pace of accumulation of growing degree days at this treatment clearly exceeded rates of accumulation found in typical field winter and spring sowings, considering the same period of crop development (Figure S1). During the experiment, average temperature exceeded 25°C half of the days. During the treatments, all pots were weighted, watered, rotated and their positions swapped every 2 days.

### Measurement of phenotypic traits

Several traits were recorded 60 days after transplant. Leaf water potential (LWP) in leaves was measured at noon using a Scholander chamber (SF-PRES-70, Solfranc Tecnologías SL, Vila-Seca, Spain). Stomatal conductance (SCo) was measured, starting at 9 am, using a leaf porometer (Decagon Devices, Pullman, WA, USA). Relative water content (RWC) was also estimated, as described in Talame et al. (2007). For each plant, three independent measurements were taken for LWP, SCo, and RWC. In addition, tiller number per plant (TN) was counted. All measurements were taken at two biological replicates.

### RNA extraction and transcriptome sequencing

Two tissues, young inflorescences and leaves were sampled at 60 days after transplant. Flag leaves were collected. In some cases, flag leaves of main tillers were too small to ensure having enough tissue for RNA extraction. In these cases, last fully expanded leaves of secondary tillers were also harvested, as a backup. Fresh material was harvested and frozen in liquid N_2_ before RNA extraction with the NucleoSpin® RNA Plant kit (Macherey-Nagel, Düren, Germany). RNA quality was assessed with a NanoDrop 2000 spectrophotometer (Thermo Scientific, Wilmington, DE, USA) and with Bioanalyzer 2100 hardware (Agilent, Santa Clara, CA, USA; average RIN: 6.7 for leaves, 8.1 for flowers). Barcoded cDNA libraries were prepared at CNAG (Barcelona, Spain) following Illumina TruSeq standard procedures, and eventually sequenced in an Illumina HiSeq2000 sequencer, using a full flow-cell, 4 samples per lane, to produce 2 × 101 bp paired-end reads. The whole dataset consisted of 2 biological replicates from greenhouse plants (2 tissues × 2 replicates × 2 genotypes), 2 biological replicates of developing inflorescences and 3 biological replicates of leaves from plants subjected to drought and well irrigated plants in the growth chamber (5 × 2 genotypes × 2 treatments). Leaf samples used for sequencing finally came from flag leaves and last fully expanded leaves in equal numbers. The results of both types of leaves were indistinguishable, and thus were treated as the same tissue.

### RNAseq data preprocessing and transcriptome assembly

Raw reads were sequentially processed with FASTQC v0.10.0 (Andrews, 2010) and Trimmomatic v0.22 (Bolger et al., 2014), discarding stretches of mean Phred score <28 and cropping the first nucleotides to ensure a per-position A, C, G, T frequency near 0.25. Only reads of length ≥80 nucleotides were kept for further analysis. Surviving reads were error-corrected with Musket v1.0.6 (Liu et al., 2013) and default parameters. Then, reads were assembled following two different procedures, *de novo* and reference-guided.

*De novo* assemblies were obtained using Trinity r2013-02-25 recommended procedures (Haas et al., 2013). First, reads from sample replicates were pooled together and *in silico* normalized, to a maximum coverage of 30. This procedure was repeated with the resulting read sets to obtain, for each genotype, a final set of normalized reads. These were used for *de novo* assembly of SBCC073 and Scarlett transcriptomes.

A reference-guided assembly (RGA) was generated with the Tuxedo pipeline (Trapnell et al., 2012). First, clean reads were mapped to the IBGSC cv. Morex assembly (Mayer et al., 2012) with Tophat2 (v2.0.9; –b2-very-sensitive, –b2-scor-min C, − 28,0 –read-mismatches 4–read-gap-length 12 –read-edit-dist 12-G 21Aug12_Transcript_and_CDS_structure.gff). This mapping procedure was performed in two steps, a first one to exclude reads with multiple mappings to the whole reference assembly (-M, -g 1, –no-discordant) and a second one to identify reads mapping unambiguously to gene coding loci (-g 2, –no-discordant, –no-mixed). Mappings were used as input for Cufflinks (v2.2.1). Individual assemblies were merged with the reference Morex assembly with Cuffmerge.

### Correction, validation and annotation of *de novo* transcriptomes

Clean reads were mapped back to the *de novo* transcriptomes using Trinity script *alignReads.pl* with Bowtie (Langmead et al., 2009). In addition, the newly assembled isoforms were mapped to Morex, Bowman, Barke WGS (Whole Genome Shotgun) assemblies (Mayer et al., 2012) and Haruna Nijo flcDNAs (Matsumoto et al., 2011) with the script *bmaux_align_fasta* from the Barleymap package (Cantalapiedra et al., 2015) (hierarchical = yes query-mode = cdna thres-id = 98 thres-cov = 10), keeping together sequences matching the same reference sequence. Sequences in each of these groups were clustered with WCD-express v0.6.3 (Hazelhurst and Liptak, 2011) using threshold = 24, which is equivalent to a 98% identity cut-off.

Presence of these isoforms in existing references was further confirmed by aligning them iteratively to additional sequence repositories. These were the Haruna Nijo genome assembly (Sato et al., 2016), genome contigs of Chinese Spring wheat (Mayer et al., 2014), barley ESTs from HarvEST assembly 36 (Close et al., 2007), the MIPS repeat database (Nussbaumer et al., 2013), and sequences from *Hordeum, Brachypodium, Triticum, Oryza* or *Aegilops* in the nt NCBI database (ftp.ncbi.nlm.nih.gov/blast/db). Alignment to Morex, Bowman and Barke WGS assemblies, and to Haruna Nijo genome and flcDNAs was repeated with a more stringent coverage threshold (thres-cov = 80). Finally, transcripts were scanned for the presence of sequencing vectors by comparison with the EMVec database (ftp://ftp.ebi.ac.uk/pub/databases/emvec/) and as a result 64 sequences were removed.

Gene annotation of assembled contigs was performed with the script *transcripts2cdsCPP.pl* (-n 50) from GET_HOMOLOGUES-EST (v 04052016, https://github.com/eead-csic-compbio/get_homologues, Contreras-Moreira et al., 2017), which uses Transdecoder (https://transdecoder.github.io/) and blastx alignments to SwissProt proteins to define CDS sequences. Clusters obtained with GET_HOMOLOGUES-EST (get_homologs-est.pl -t 0 -M -S 96 -A –L), requiring percentage sequence identity >96, were used to obtain reciprocal correspondences between transcripts from SBCC073 and Scarlett assemblies. PFAM domains in translated CDS sequences were also annotated (*get_homologs-est.pl* –D).

### Analysis of gene expression

Differential expression contrasts were performed for each genotype, tissue and treatment; both for isoforms and genes. For this purpose, we compared three different pipelines.

For the first one, estimation of expression levels of isoforms and genes was done with RSEM v.1.2.11 (Li and Dewey, 2011), using Bowtie2 (Langmead and Salzberg, 2012) and otherwise default parameters. RSEM “expected counts” were used as input for differential expression analyses with the “glm” functions of the R (R Development Core Team, 2008) Bioconductor package edgeR v3.8.6 (Robinson et al., 2010) (false discovery rate function “BH” set to 0.001). A minimum CPM (counts per million) of 0.4, equivalent to around 10 RSEM “expected counts” based on a linear regression (R-square = 1, intercept ~ 0, slope = 25), was required in at least half of the samples to include an isoform or a gene in the analysis.

A second method relied on kallisto v0.42.5 (Bray et al., 2016) to obtain “expected counts” and to generate 100 bootstrap samples for each replicate, followed by test for differential expression with sleuth v.0.28.0 Wald test (Pimentel et al., 2016), using the previously generated bootstrap samples.

For the third method, Cuffquant and Cuffdiff v.2.2.1 (Trapnell et al., 2013) were used to test differential expression, with FDR 0.05, on the RGA transcripts.

Principal component analyses (PCA) of the resulting expression estimates from kallisto were done with the function PCA from R package FactoMineR 1.29 (Lê et al., 2008). Correlation analysis was performed using the R package corrplot 0.73 (Wei and Simko, 2014).

### RT-qPCR validation

Reference genes for calculating relative expression were either searched in the literature or selected from our RNAseq data. The latter were those with the smallest coefficient of variation of expression values across samples, among isoforms not reported as differentially expressed (DE) by edgeR. DE isoforms to be checked with RT-qPCR were chosen randomly from bins covering the range of edgeR logFC. All the selected DE isoforms had TPM (transcripts per million) >1. Primers for both reference genes and DE isoforms were designed with Primer Express 3.0 (Applied Biosystems). Conservation of the target sequences was checked in both SBCC73 and Scarlett isoforms. Whenever possible, one of the primers of the pair was set over an exon-exon junction and toward the 3′ end.

The same DNase I-treated RNA samples used for RNAseq were utilized for the RT-qPCR assays. First strand cDNA synthesis was made from 2 μg of total RNA to a final volume of 40 μl containing oligo(dT)20 for priming and SuperScript III Reverse Transcriptase (Invitrogen, Cat.No. 18080-044). All the RT-qPCR reactions were performed in an ABI7500 (Applied Biosystems, Foster City, CA, USA) with the following PCR profile: 95°C 10 min pre-denaturation step; 95°C 15 s denaturation and 60°C 50 s annealing (40 cycles), followed by a melting curve 60°–95°C default ramp rate. The efficiency of primers was obtained from calibration curves with 1:5 dilution series and at least 4 points fitted in a linear regression with R-square over 0.99. We used NormFinder (Andersen et al., 2004) to analyze the stability value of the reference genes. Relative change of expression was calculated according to Pfaffl (2001), but using the geometric mean of three reference genes as normalization factor (Vandesompele et al., 2002).

### Functional annotation of differentially expressed isoforms

Software CPC (Kong et al., 2007) was used to tag DE isoforms as coding or non-coding, and to obtain Uniref90 best hits. In addition, contained CDS sequences were deduced and PFAM protein domains annotated, as explained earlier for all the isoforms of each transcriptome. GO terms for each DE isoform were obtained with in-house script barleyGO (http://www.eead.csic.es/compbio/soft/barleyGO.tgz). Enrichment tests for PFAM domains and GO terms were performed in R using the Fisher exact test (*p* < 0.01). For the GO terms, we used the R package topGO (Alexa and Rahnenfuhrer, 2016).

DE isoforms were searched in metabolic pathways databases, including KEGG (Kanehisa et al., 2016), PlantReactome (Tello-Ruiz et al., 2016), and PlantCyc (Plant Metabolic Network, 2016). For KEGG, we obtained the list of genes of *Oryza sativa* (“osa”), from which we retrieved Orthology identifiers and pathways. DE isoforms were aligned to those genes with blastn (-perc_identity 75 –num_alignments 1), discarding hits with low query coverage in the alignment (“qcovs” < 70). PlantReactome (file “gene_ids_by_pathway_and_species.tab”) was explored with Morex gene identifiers to obtain the pathways involved in differential expression. The gene identifiers were derived from mappings of *de novo* assemblies to the Morex reference genome from the validation step using the Barleymap package, as explained above. In the case of PlantCyc, we obtained the blast set “plantcyc.fasta” and enzymes annotation (“PMN11_June2016/plantcyc_pathways.20160601”), and used a custom script to match annotated enzymes with blastx (*e*-0.00001–num_alignments 1), filtering hits with percentage identity ≥75. Enzymes and pathways were grouped in broader categories manually, by merging their textual descriptions in KEGG and PlantCyc.

### Comparison with related studies

The literature was surveyed to obtain protein and transcript sequences which had been previously associated with response to water deprivation in barley. These drought-related sequences were aligned with Blast[p|x] to genes from the Haruna Nijo genome assembly, which allowed mapping them to their corresponding DE isoforms from this study.

### Clustering and identification of cis-regulatory elements of co-expressed genes

DE isoforms were clustered based on their TPM values (from kallisto). Distance between each pair of isoforms was calculated with Pearson correlation. This metric was weighted with Euclidean distance, under the hypothesis that isoforms sharing their expression pattern, but differing in magnitude, might have promoters which could be overlooked when clustered together with Pearson correlation only. These distances were used to perform hierarchical clustering (R package hclust, method = “complete”). To declare the final number of clusters, the dendrogram was pruned when 95% of clusters had an internal average distance below 0.001% of the initial average distance of all DE isoforms.

The following procedure was used to recover promoter sequences corresponding to the genes present in the expression clusters. DE isoforms from each cluster were mapped to transcripts from the Morex WGS assembly (Blastn -perc_identity 98). For each cluster containing 10 or more genes, repeat-masked promoter sequences (−1,000, +200 nucleotides around TSS) were retrieved from the RSAT::Plants server (http://plants.rsat.eu, version Hordeum_vulgare.082214v1.29) (Medina-Rivera et al., 2015). As negative controls, promoter sequences were retrieved from randomly generated gene clusters of the same size. Enrichment in GO terms and motif discovery with oligo-analysis and dyad-analysis were performed following the protocol of (Contreras-Moreira et al., 2016). Motif scores within upstream regions of co-expressed genes and their orthologous genes in *Brachypodium distachyon* reference (v1.0.29) (International Brachypodium Initiative, 2010), were obtained with the program matrix-scan from RSAT::Plants. These scores were also calculated for motifs generated by permutation of the bases of each discovered motif. Therefore, two types of evidences were used to assess the reliability of discovered motifs: (i) their statistical significance compared to the negative controls, and (ii) their matrix-scan scores compared to the scores of permuted motifs. Discovered motifs were annotated by comparison to plant regulatory motifs in the footprintDB repository (Sebastian and Contreras-Moreira, 2014). The highest scoring motif, in terms of footprintDB “Ncor” score, was selected as the best hit. The full report on the promoter analysis, including source code, is available at http://floresta.eead.csic.es/rsat/data/barley_drought_clusters.

Finally, deduced peptide sequences of DE isoforms annotated as transcription factors with iTAK (http://bioinfo.bti.cornell.edu/cgi-bin/itak/index.cgi), were used to predict their putative DNA-binding motifs with footprintDB.

## Results

### Growth of Scarlett and SBCC073 plants subjected to drought

**Figure 1:**
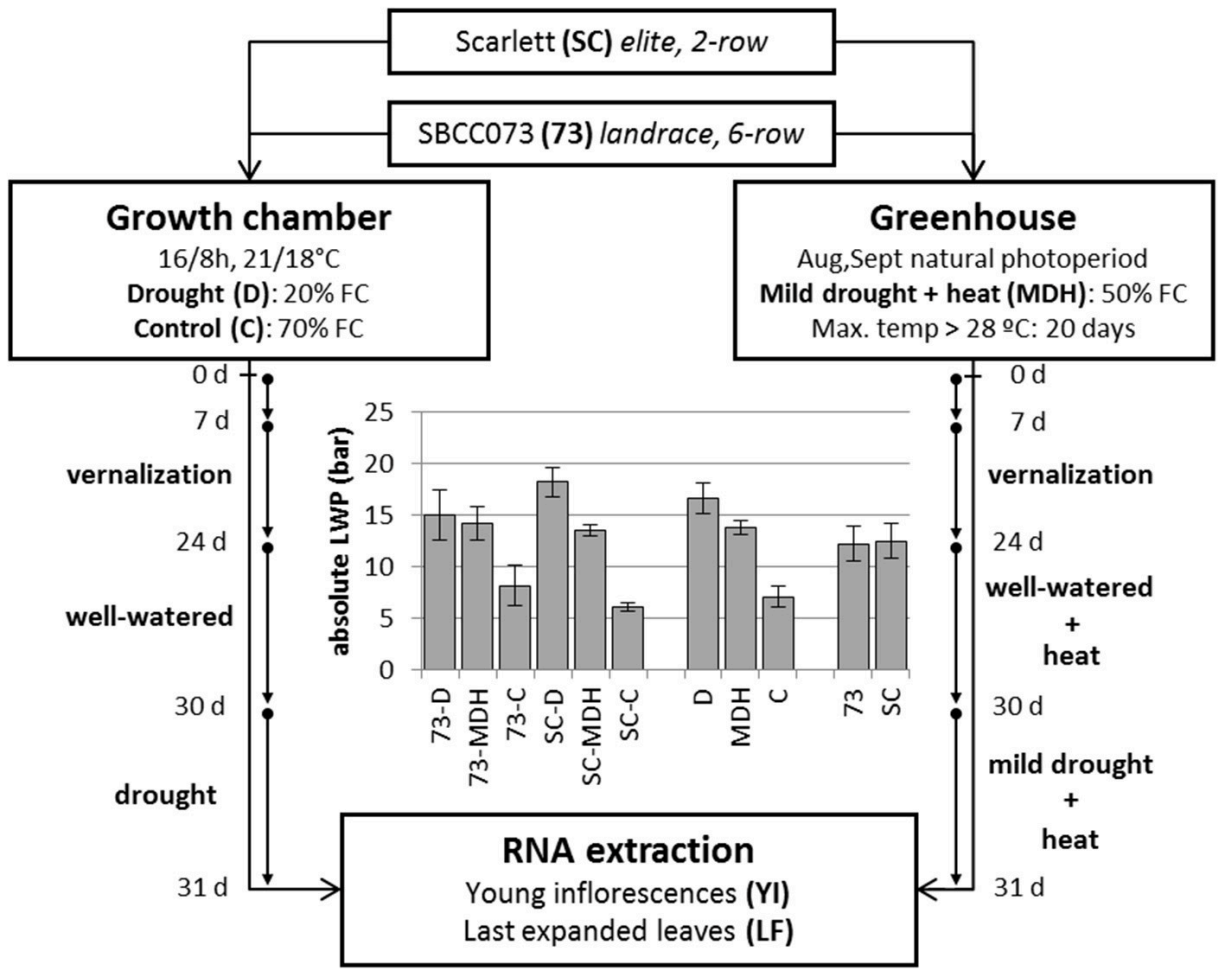
**Design of stress treatments, and leaf water potential patterns**. SBCC073 (73) and Scarlett (SC) plants were placed in a growth chamber and in a greenhouse, after 24 days (24 d) of vernalization. Drought treatments lasted 30 days (30 d), after 30 d of normal irrigation. Growth chamber plants were either watered to 70% FC (control, C) or instead 20% FC (drought, D). Greenhouse plants were subjected to heat stress, for 30 d, followed by mild drought (50% FC) combined with heat stress (MDH), for another 31 d. The bar plot shows average ± SEM absolute leaf water potential (LWP).

Two different experiments were set up, in which plants were placed in a growth chamber or in a greenhouse. The growth chamber was kept at strictly controlled environmental conditions, whereas the greenhouse underwent a natural photoperiod (August–September, 2012, starting with 14 h 23 min and ending with 11 h 46 min daylight, http://www.fomento.gob.es/salidapuestasol/2012/Zaragoza-2012.txt) and controlled temperature, surpassing typical temperatures expected under field conditions (Figure S1), but more variable irradiation and humidity. Both daytime and night temperatures in the greenhouse were higher than in the growth chamber, whereas relative humidity was similar on average (Figure S2). In both settings, water stress was imposed after initiation of the stem elongation stage. Growth chamber plants were watered in order to stay at 70% field capacity (FC) (controls, C), or instead subjected to reduced irrigation until reaching 20% FC (drought, D). Plants in the greenhouse were irrigated to an intermediate 50% FC (mild drought and heat, MDH). These experiments are outlined in Figure 1.

Daily loss of water, based on the weights of pots, was largest in C plants, intermediate under MDH and lowest under D (Figure S3). The same trend was observed for leaf water potential (LWP), summarized in Figure 1. LWP was proportional to the three imposed water regimes, with plants subjected to drought (D and MDH) showing larger absolute LWP that those well-watered. The largest absolute value corresponded to Scarlett plants under D, in which SBCC073 plants had values comparable to those of both SBCC073 and Scarlett plants under MDH. Likewise, minimum values for stomatal conductance (SCo) were recorded for plants under D (Table 1). However, the largest SCo was found under MDH. Relative water content (RWC) was lowest for plants under D, in both genotypes, whereas under MDH, it was closer to that of C plants in SBCC073, and closer to that of plants under D in Scarlett. Tiller number (TN) was also affected by water deprivation, being larger in C than under D, both in SBCC073 and Scarlett. Under MDH, similarly to the RWC observations, TN was less affected in SBCC073 than in Scarlett.

**Table 1:** **Physiological measurements of plants in the drought experiments**.

**Treatment**	**LWP (bar)**	**SCo (mmol/m^2^s)**	**RWC**	**TN**
**SBCC073**				
C	8.09	33.57	0.94	13
MDH	14.10	40.93	0.97	11
D	14.95	23.02	0.82	8
**SCARLETT**				
C	6.00	12.45	0.92	16
MDH	13.47	39.00	0.85	5
D	18.15	0.25	0.87	11

*Treatments corresponded to control (C) and drought (D) in the growth chamber, at 70 and 20% field capacity (FC), respectively; whereas greenhouse plants were kept at mild drought and heat (MDH, 50% FC). Physiological and morphological measurements were absolute leaf water potential (LWP), stomatal conductance (SCo), relative water content (RWC) of leaves, and tiller number per plant (TN)*.

### Assembly and validation of Scarlett and SBCC073 transcriptomes

Sequencing of cDNA libraries, derived from leaf (LF) and young inflorescence (YI) transcripts, yielded 1.18 billion paired-end reads. From this dataset, we assembled separate *de novo* transcriptomes for Scarlett and SBCC073, as well as a reference-guided assembly (RGA) (Figure S4).

**Figure 2:**
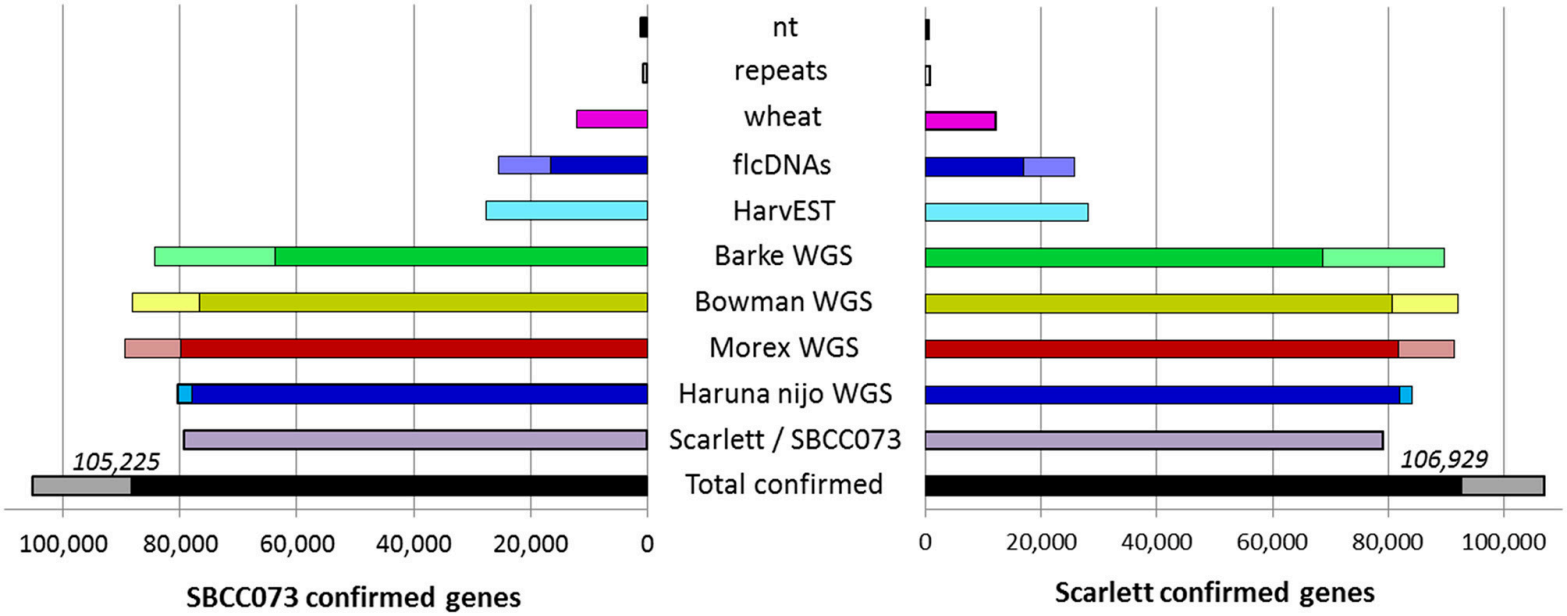
*****De novo*** assembled genes confirmed in existing barley references**. Bars indicate the number of assembled genes of landrace SBCC073 (left) and cultivar Scarlett (right) which were confirmed by alignment to each other, and to several sequence repositories of barley and wheat (for list, see text). The total number of genes confirmed for each of the two assemblies is also shown (bottom black/gray bars). The alignments required 98% identity and a minimum alignment query coverage of either 10% (whole bars) or 80% (fraction of bars filled with a darker color).

The *de novo* assemblies yielded similar numbers and lengths of isoforms for both genotypes (Table 2). These sets, with 103,623 genes in SBCC073 and 113,962 in Scarlett, were comparable but larger than the annotated gene sets for the Morex cultivar (Mayer et al., 2012), with 75,258 high and low confidence genes, and with the results from the RGA (75,204 genes). Validation and correction of the *de novo* isoforms was performed in three stages. First, the clean reads were mapped back to the assembled transcripts, to compute the fraction of well aligned pairs of reads (both reads mapped, correct orientation and insert size), which was near 83% for both cultivars. Second, *de novo* subcomponents were revised for re-clustering. This requires some explanation. Whereas RGA contigs are isoforms associated to known genes from the reference, *de novo* assembly generates contigs which are isoforms clustered in so called subcomponents. In some cases, these subcomponents accumulate closely related sequences, for instance from paralogous genes or expressed pseudogenes, which should be separated. Therefore, this second step consisted in re-clustering isoforms from subcomponents to genes, by alignment to annotated references (see Methods), and assigning them to different loci when appropriate. The final number of genes in the *de novo* assemblies was 112,923 in SBCC073 and 123,582 in Scarlett. Third, the isoforms were matched to a variety of genomic and transcriptomic sequence repositories of barley, wheat and other grasses. In total, 93% of SBCC073 and 87% of Scarlett genes could be confirmed. These sequence comparisons are further illustrated in Figure 2. Note that at least 10% alignment coverage was required in all cases. Further, the alignment against Morex, Barke, Bowman and Haruna Nijo was repeated, with a more strict minimum coverage of 80%. This test confirmed that 88,293 (78% of SBCC073) and 92,713 (75% of Scarlett) genes map with high confidence to previously reported barley sequences.

**Table 2:** **Statistics of ***de novo*** and reference-guided assemblies**.

**Assembly**	**Isoforms**	**Genes**	**N50**	**Mean length**	**Annotated (%)**	**SwissProt**	**Transdecoder**
SBCC073	303,872	112,923	2,589	1,603	195,184 (64%)	87,145	108,039
Scarlett	307,168	123,582	2,537	1,538	175,779 (57%)	84,310	91,469
RGA	146,427	75,204	4,085	2,512	96,107 (66%)	19,513	76,594

*Rows correspond to either de novo assemblies (SBCC073 and Scarlett) or reference-guided assembly (RGA). The upper part of the table shows the number of isoforms and genes, as obtained from the assembler, along with statistics on length of isoforms (N50 and mean length). The bottom half shows the number and percentage of annotated isoforms, and whether this annotation was obtained from alignment to SwissProt database or by CDS de novo prediction with Transdecoder*.

### Analysis of gene expression

Clean paired-end reads were mapped back to SBCC073 and Scarlett assemblies, to estimate expression counts for each transcript. These estimates were subsequently used to identify DE tags (genes and isoforms) between stressed treatments and C, for each tissue and genotype. For this purpose, we compared three different pipelines, which rely on different software for each of the two steps: RSEM-edgeR, kallisto-sleuth and Cuffquant-Cuffdiff. In addition, a set of isoforms from YI were randomly chosen to test their expression by RT-qPCR, using genes selected from the literature and from our RNAseq expression data as references (Table S1).

**Figure 3:**
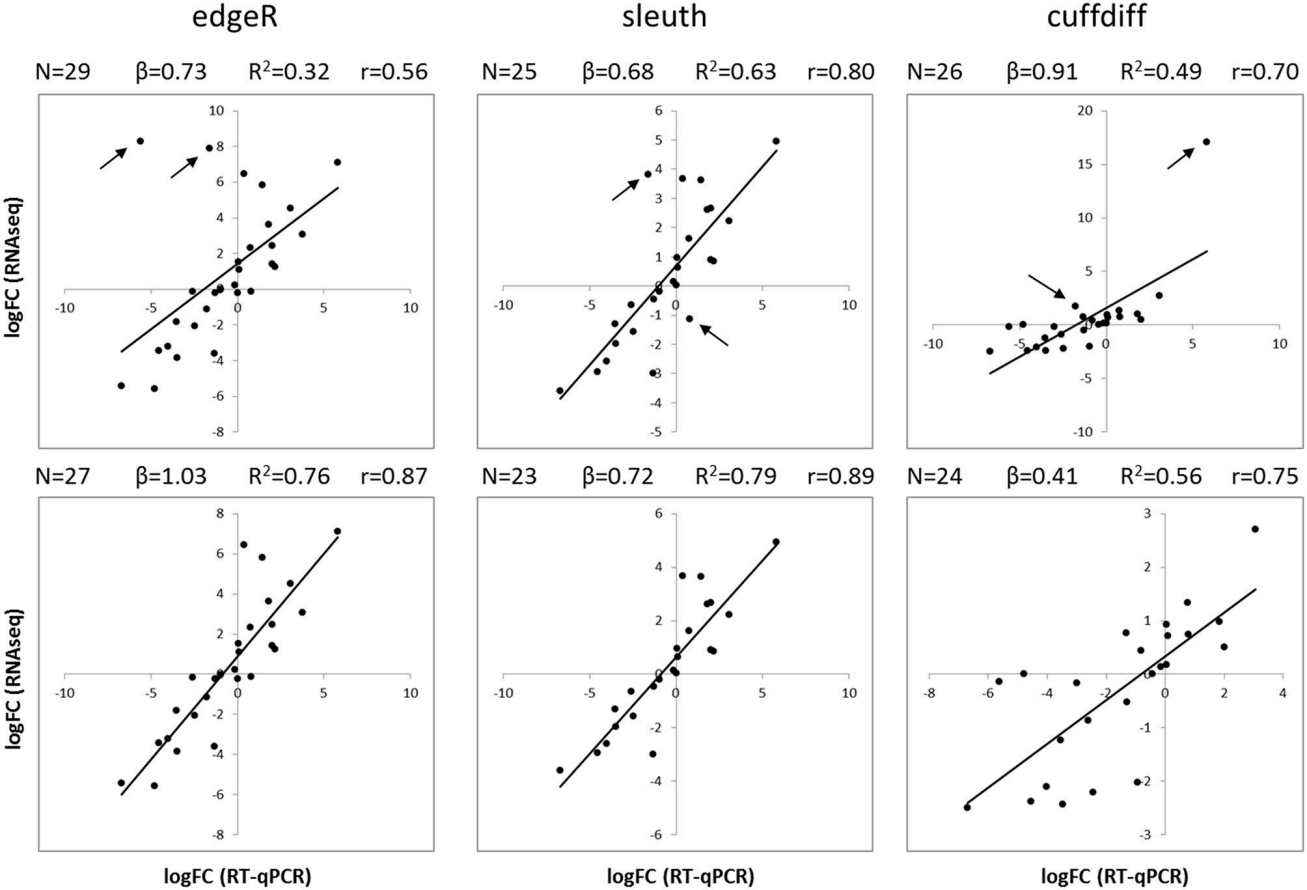
**Comparison of RT-qPCR and RNAseq gene expression results**. Scatterplots show the logFC of isoforms obtained with RT-qPCR (horizontal axis) and with RNAseq (vertical axis). LogFC values from RNAseq were obtained with three different analysis methods: edgeR (left), sleuth (center) and Cuffdiff (right). Plots on the top show all available data, whereas plots on the bottom show data after removing the two most scattered data points (black arrows). Black lines correspond to a linear regression. N, number of data points; β, slope of regression; R^2^, coefficient of determination; r, Pearson correlation coefficient.

The results of differential expression computed with kallisto-sleuth had the best agreement with those of RT-qPCR (Figure 3). The outcome of the RSEM-edgeR pipeline was comparable to kallisto-sleuth after discarding a few outliers. Moreover, PCA and clustering of samples, using expression estimates from kallisto, showed good correlation between replicates (Figures S5, S6). When the expression estimates, obtained with the three methods, were directly compared, RSEM-edge and kallisto-sleuth showed the best agreement (Figures S7-S9 and Table S2). In order to reduce false positives, final DE tags were obtained from the intersection between those two methods.

**Figure 4:**
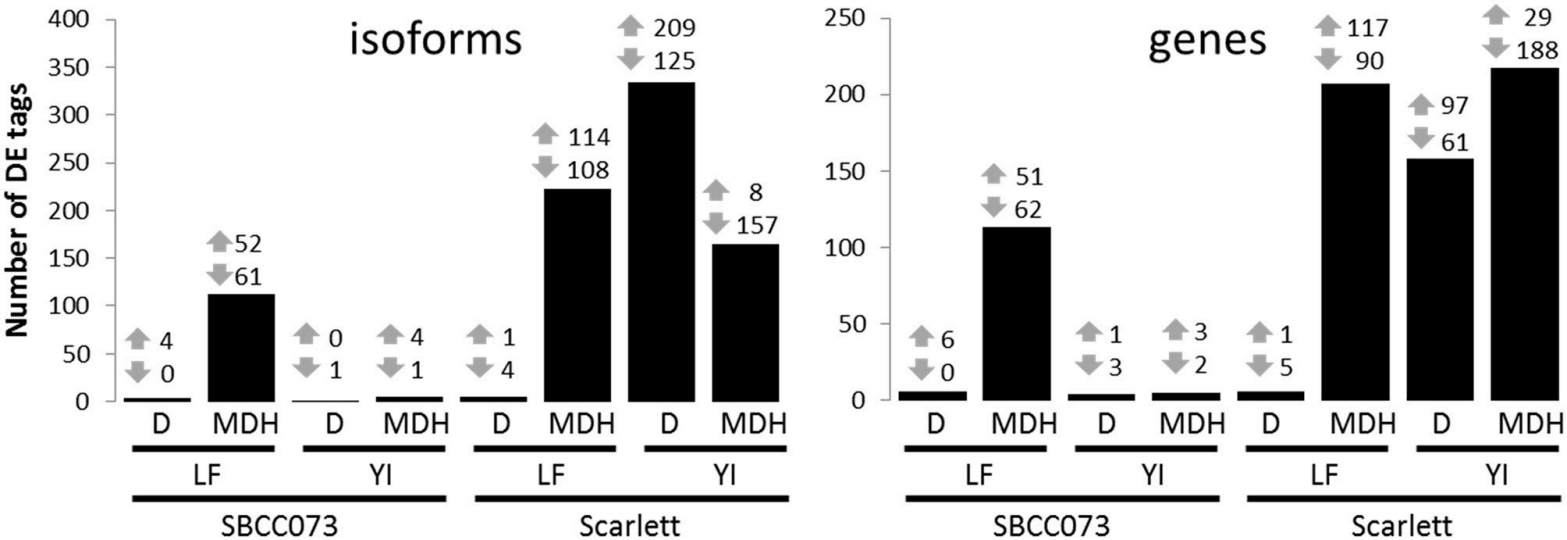
**Number of differentially expressed isoforms and genes**. Number of up-regulated (up arrows) and down-regulated (down arrows) differentially expressed tags (isoforms, left; genes, right), for each contrast. Bars show the sum of both induced and repressed tags. LF, leaves; YI, young inflorescences; D, drought treatment; MDH, mild drought and heat treatment.

Overall, the response differed between genotypes in YI, and between treatments in LF (Figure 4). Under D, we found almost no response in SBCC073, either in YI or LF samples, whereas in Scarlett, YI samples had many DE tags. On the contrary, abundant changes in gene expression were observed under MDH, with the exception of YI from SBCC073, which remained mostly unaltered. Regarding the proportion of up-regulated tags over total DE tags, in LF under MDH it was close to 50%, in both genotypes, whereas in YI from Scarlett plants it increased under D (62.6% in isoforms, 61.4% in genes) and decreased dramatically under MDH. There was high agreement between DE genes and DE isoforms in all contrasts, although some DE genes were different to those found when analyzing isoforms (Table S3). On the other hand, common DE tags between different contrasts were negligible, with the exception of LF under MDH, in which Scarlett and SBCC073 shared a low but sizable fraction (Figure S10).

We explored the cases in which there were several isoforms associated to a single gene differentially affected by the treatments. First, we studied those genes which had more than one isoform in the same contrast (Data Sheet 2). For example, in LF from Scarlett under MDH there were 54 DE isoforms corresponding to 21 genes, ranging from 2 to 6 isoforms per gene. Out of these, just 2 isoforms from a single gene showed opposite directions of change in gene expression. As in that example, for all the other contrasts, the direction of change was the same for most groups of isoforms. The outstanding exception was YI from Scarlett under D, with more than one third of isoforms showing different induction/repression. This suggests that the transcriptional landscape of YI from Scarlett was dramatically affected by D. Second, we assessed the function of isoforms from a single gene, which were differentially expressed in different contrasts (Data Sheet 3). In SBCC073, we found only 4 isoforms, from a single gene annotated as CCA1/LHY (see below), which were all of them induced. Of these, 2 of them occurred in YI and LF under MDH, and 2 in YI only. All 4 isoforms were annotated as coding, putative splice variants. In Scarlett, we found 61 isoforms, associated to 24 genes, 9 of them with isoforms with opposite directions of change in gene expression. For example, a gene annotated as alpha-glucan water dikinase had 9 isoforms of which 3 were induced in YI and 6 were repressed in LF, under MDH. These two groups differed in functional annotation. Moreover, a gene annotated as beta-glucosidase showed 4 DE isoforms under MDH, one induced in LF and 3 repressed in YI. Also, we observed a transcript likely encoding a protein kinase for which one isoform was induced in YI under D and another one was induced in LF under MDH.

Overall gene expression changes (number of DE tags and cumulative logFC from each contrast) were compared with the physiological measurements. Some large correlations were obtained (Table S4), although these results must be considered with care due to the small sample size. For LWP, we found a positive correlation with YI overall logFC of isoforms (*r* 0.97, *p*-value 0.03) and number of DE tags (*r* 0.99, *p*-value 0.01). SCo exhibited strong positive correlation with gene expression changes in LF (ranges: *r* 0.95–0.98, *p*-values 0.05–0.02).

DE isoforms were annotated combining different strategies, as described in Materials and Methods. The main annotation results are detailed in the following sections, whereas the complete results are provided in Data Sheet 4.

### Differentially expressed isoforms in leaves under drought

As explained in the previous section, just a few isoforms were DE in LF under D. In both genotypes, we found an up-regulated isoform encoding a polyamine oxidase, involved in spermine and spermidine degradation. In addition, an isoform corresponding to a chlorophyll apoprotein from photosystem II was down-regulated in Scarlett. However, this change was not observed in SBCC073, which instead showed induction of transcripts of three proteins, namely ABA/WDS (abscisic acid/water deficit stress) induced protein, ribonuclease T2 and calcineurin-like phosphoesterase. Other DE isoforms were annotated as non-coding or of unknown function.

### Differentially expressed isoforms in leaves under mild drought and heat

There were more DE tags in LF under MDH, and involved a more diverse array of gene functions than under D. The same polyamine oxidase induced in LF under D was also observed up-regulated in Scarlett under MDH. Intriguingly, in SBCC073 we found up-regulated a transcript encoding a spermidine synthase.

**Figure 5:**
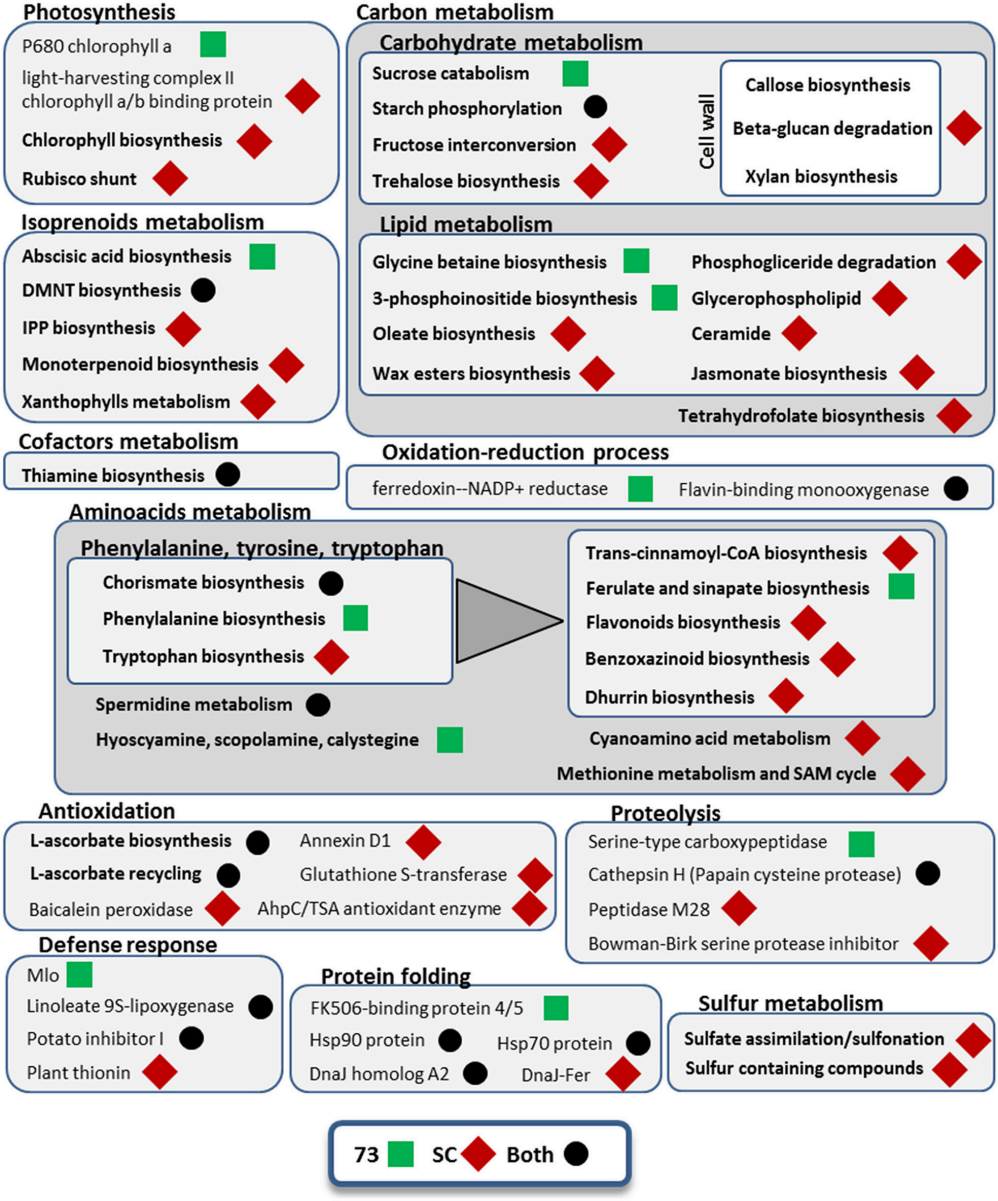
**Metabolic pathways and cellular processes with differentially expressed isoforms from leaves under mild drought and heat**. Metabolic pathways, cellular processes and proteins with differentially expressed isoforms are grouped into more general processes, within boxes. Bold categories include several differentially expressed isoforms from a given pathway or process, whereas non-bold names are from specific proteins. Green squares represent processes affected only in SBCC073 (73) plants, whereas red diamonds indicate those altered only in Scarlett (SC). Processes and proteins with changes in gene expression in both genotypes are marked with a black circle. A triangle links the metabolism of aromatic amino acids with downstream pathways of secondary metabolites obtained from them.

Some GO terms were enriched in both genotypes, including “phosphorelay signal transduction system,” “pyrimidine-containing compound biosynthesis process,” “response to temperature stimulus,” “response to water deprivation” and “thiamine biosynthetic process” (Data Sheet 5). Other pathways and cellular processes involved in the responses of both genotypes were starch phosphorylation, chorismate biosynthesis, L-ascorbate biosynthesis and recycling, DMNT biosynthesis (a volatile homoterpene), and other proteins involved in protein folding, proteolysis and defense response (Figure 5). We also found in both genotypes up-regulation of isoforms annotated as CCA1/LHY MYB-related TF (Table S5). Moreover, we found another DE gene annotated as MYB-related TF in both genotypes, which is similar to *Arabidopsis thaliana* TCL2, and an additional uncharacterized MYB-related TF in SBCC073 only. At the same time, down-regulation of other genes related with circadian rhythm was detected, like adagio-like protein 3 and a PRR1 (HvTOC1) transcription regulator. In SBCC073, we found also down-regulation of another circadian clock related gene, annotated as APRR3. Another gene up-regulated in both genotypes was annotated as protein kinase CIPK9. Regarding transporters, repressed transcripts encoding aquaporins were noticed in both genotypes. There were a few other protein domains regulated in both genotypes, most of them repressed.

Differences between genotypes were also seen among DE transcripts in LF under MDH. For instance, in SBCC073 there was enrichment of terms such as “actin filament-based movement,” “ammonium ion metabolic process” and “defense response by cell wall thickening,” while in Scarlett a greater variety of response-related terms were obtained, such as “response to abscisic acid,” “response to bacterium,” “response to ethylene,” “response to hydrogen peroxide” or “response to wounding” (Data Sheet 5). Also, DE isoforms related to glycine betaine biosynthesis and to abscisic acid (ABA) biosynthesis were seen in SBCC073, whereas trehalose biosynthesis was involved in the response of Scarlett LF to MDH (Figure 5). Moreover, isoforms involved in cell wall, epidermis (wax esters) and membrane lipids (glycerophospholipids, ceramide) metabolism were up-regulated in Scarlett but not present among SBCC073 DE isoforms. This was also the case of some defense response metabolic pathways (benzoxazinoids and dhurrin biosynthesis), xanthophylls metabolism, several antioxidation related proteins (like baicalein peroxidase or glutathione S-transferase) or sulfur metabolism related proteins. We also found differences among TFs and protein kinases (PKs) (Table S5). For instance, CIPK17 and a C2C2-Dof TF, whose best SwissProt hit is Arabidopsis protein CDF2, were up-regulated, and an AP2/ERF-AP2 TF (related to *Brassica napus* BBM2) down-regulated, all in SBCC073. Instead, repression of a TUB TF, similar to *O. sativa* subsp. *japonica* TULP7, and induction of both a bZIP TF and a jasmonate ZIM TIFY TF, the latter related to *O. sativa* subsp. *japonica* TIFY6B, was noticed in Scarlett. Besides aquaporins, already mentioned, DE isoforms related to transport processes were different between genotypes, being more abundant in Scarlett. These included lipid transfer proteins, phosphate, potassium, triose-phosphate, adenine, vacuolar amino acid and ABC transporters, and a repressed NUCLEAR FUSION DEFECTIVE 4 (NFD4) protein.

### Differentially expressed isoforms in young inflorescences in SBCC073

In YI, the transcriptional responses were markedly different between genotypes, with only minor responses in plants of genotype SBCC073 under both treatments. Indeed, a single down-regulated transcript was identified in SBCC073 under D, annotated as Pollen Ole e 1 allergen/extension. Under MDH, a repressed isoform was annotated as “non-coding,” whereas four up-regulated isoforms corresponded to CCA1/LHY.

### Differentially expressed isoforms in young inflorescences in Scarlett

**Figure 6:**
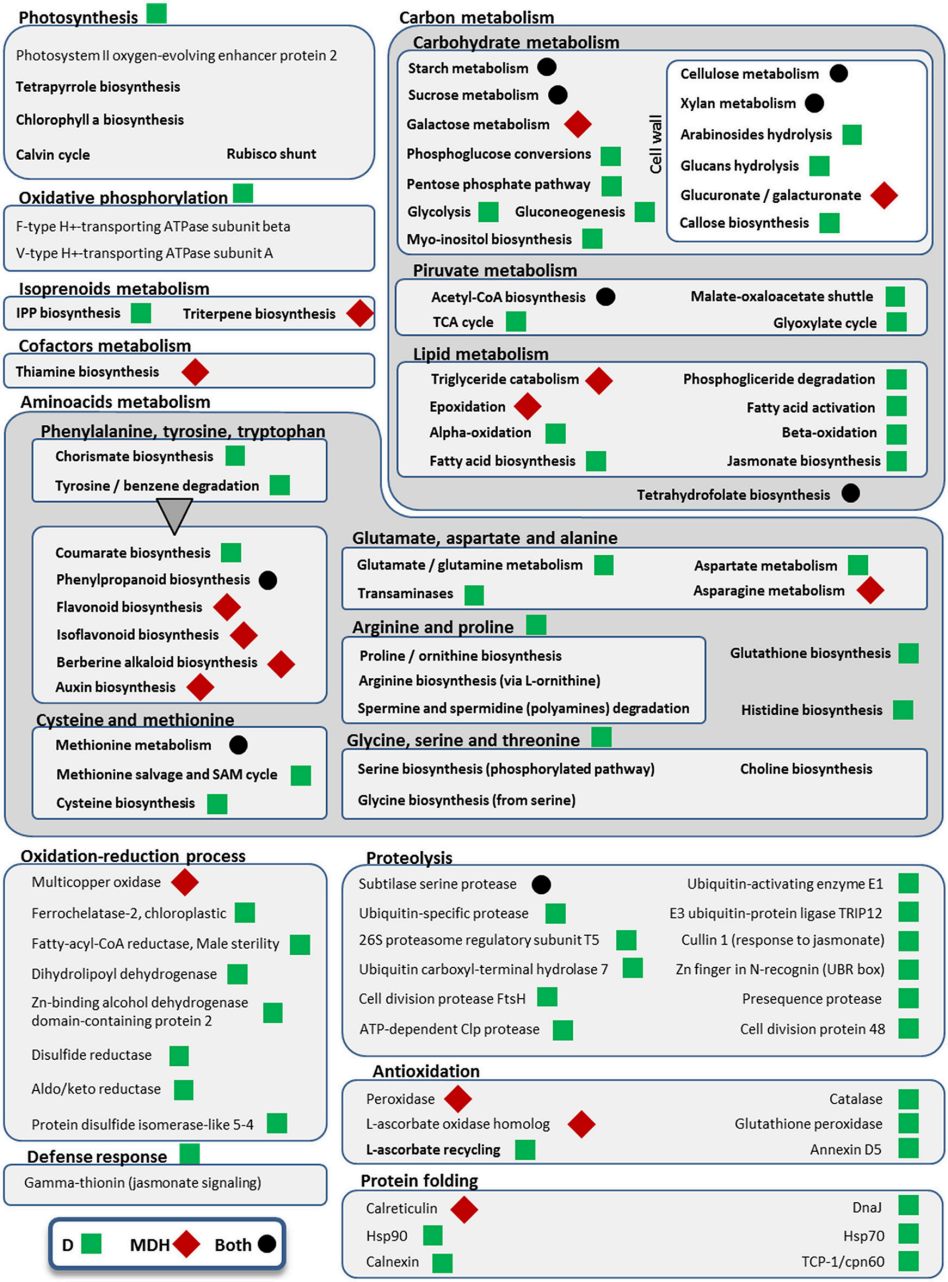
**Metabolic pathways and cellular processes with differentially expressed isoforms from Scarlett young inflorescences**. Metabolic pathways, cellular processes and proteins with differentially expressed isoforms are grouped into more general processes, within boxes. Bold categories include several differentially expressed isoforms from a given pathway or process, whereas non-bold names are from specific proteins. Green squares point out processes altered only under drought (D), whereas red diamonds indicate processes affected only in the mild drought and heat experiment (MDH). Processes and proteins with changes in gene expression in both treatments are marked with a black circle. A triangle links the metabolism of aromatic amino acids with downstream pathways of secondary metabolites obtained from them.

In contrast with what was seen in SBCC073, YI from Scarlett showed abundant gene expression changes. Enriched GO terms found both under D and under MDH were scarce (Table 3), including cell wall-related processes “beta-glucan biosynthetic process,” “lignin metabolic process,” “phenylpropanoid metabolic process,” and “cell wall organization or biogenesis,” and others like “response to carbon dioxide” and “sucrose metabolic process.” Other shared DE tags included isoforms involved in tetrahydrofolate biosynthesis and a subtilase serine protease (Figure 6). Among DE TFs in YI, we found B3-ARF isoforms (Auxin response factors with B3 and PB1 domains) induced under both treatments (Table S6). However, reciprocal alignment revealed that they belong to different genes (blastn, alignment coverage 48% and percentage of identity 63%). The most similar protein of the isoform in the D treatment was ARF21, also known as OsARF7b, whereas the closest homolog of the isoform found under MDH was ARF11.

**Table 3:** **Gene Ontology terms enriched in Scarlett young inflorescences**.

**MDH and D**	**MDH only**
Beta-glucan biosynthetic process	Cellulose biosynthetic process
Lignin metabolic process	Xylan biosynthetic process
Phenylpropanoid metabolic process	Plasmodesmata-mediated intercellular transport
Response to carbon dioxide	Mucilage extrusion from seed coat
Sucrose metabolic process	Flavonoid biosynthetic process
Cell wall organization or biogenesis	Mitotic chromosome condensation
**D only**	
ARF protein signal transduction	Growth
Aspartate family amino acid biosynthetic process	Hydrogen peroxide catabolic process
ATP generation from ADP	L-alanine catabolic process, by transamination
ATP hydrolysis coupled proton transport	L-phenylalanine catabolic process
Callose deposition in cell wall	Methionine biosynthetic process
Carbohydrate catabolic process	NADP metabolic process
Cell wall thickening	ncRNA transcription
Cellular response to starvation	Pentose-phosphate shunt
De-etiolation	Polycistronic mRNA processing
Embryo development ending in seed dormancy	Positive regulation of embryonic development
Ethylene biosynthetic process	Positive regulation of ribosome biogenesis
Glucose metabolic process	Primary root development
Glycerol catabolic process	Protein import into chloroplast stroma
Pyruvate metabolic process	Starch metabolic process
Response to metal iron	Sulfur amino acid biosynthetic process
Response to hormone	Translation elongation
Response to osmotic stress	Tricarboxylic acid metabolic process
S-adenosylmethionine biosynthetic process	Triglyceride mobilization
Seed development	Wax biosynthetic process

*The upper left section shows the GO terms enriched in both experiments (MDH: mild drought and heat; D: drought). The upper right section shows the GO terms enriched only under MDH. The bottom section shows the GO terms enriched only among differentially expressed isoforms under D*.

Besides B3-ARF TFs, only a few other isoforms were up-regulated in Scarlett YI under MDH, corresponding to an elongation factor EF-1, a DNA topoisomerase, a kinesin motor domain, CCA1/LHY, and a condensing complex subunit protein. All the others were down-regulated, whose enriched GO terms included “cellulose biosynthetic process,” “xylan biosynthetic process,” “flavonoid biosynthetic process,” “mitotic chromosome condensation,” “plasmodesmata-mediated intercellular transport” and “mucilage extrusion from seed coat” (Table 3). Other differences with respect to the D treatment were the involvement of enzymes from thiamine biosynthesis, triglyceride catabolism, epoxidation, berberine alkaloid biosynthesis or auxin biosynthesis (Figure 6). Among repressed isoforms related with transporters, we found sugar and lysine-histidine transporters, a PRA1 family protein B2 (a protein family related to regulation of vesicle trafficking, (Kamei et al., 2008), and several ABC transporters (Table S6; Data Sheet 5). Other proteins (and protein domains) which were found DE only under MDH included an expansin-B3, a putative cell wall protein, a PMR5/Cas1p, and several germin-like proteins.

Under D, Scarlett YI showed almost twice as many induced than repressed isoforms. The number of enriched GO terms was greater than for all the other contrasts (Data Sheet 5), including numerous enriched processes (Table 3) and metabolic pathways (Figure 6), related with responses to abiotic stress (cell wall thickening, biosynthesis of wax, triglyceride mobilization, expansin-A7), development (seed, embryo and root development), central metabolism (starch, glucose, pyruvate, many amino acids, fatty acids biosynthesis, activation and beta-oxidation), hormones (ethylene, jasmonate), energy (ATP and NADP metabolism related proteins, F and V-type H+-transporting ATPases), nucleic acids and proteins metabolism, antioxidation, proteolysis, protein folding, numerous proteins involved in transport and vesicle trafficking (Table S6), tRNA synthetases, an up-regulated MADS-MIKC TF whose best hit in SwissProt is *O. sativa* subsp. *japonica* MADS6, several PKs (like CIPK30) and phosphatases (like phosphoinositide phosphatase SAC7), proteins involved in interactions and signal transduction (SNF2, ASPR1 topless-related protein 1, 14-3-3 protein epsilon, CypP450), cytoskeleton proteins (tubulin, myosin, fimbrin and villin domains), and even processes related with photosynthetic tissues, like biosynthesis of chlorophyll a or tetrapyrrole, or induction of a Rubisco activase.

All these evidences indicate that responses to D and MDH of Scarlett YI were different, and that reproductive tissues were undergoing large gene expression changes, especially under D.

### Comparison with related studies

We surveyed the literature reporting genes and proteins expressed in barley in response to water deprivation. The goal was to compare those sequences to the DE transcripts identified in this work. The studies listed in Table 4 include 5 microarray experiments, 8 based on proteomics, 1 RNAseq study, 1 QTL work, 1 surveying expression QTL and 1 meta-analysis. Most of them focused on barley plants under drought, with a few exceptions. The work “matsumoto2014” surveyed responses to desiccation, salt stress and ABA. In addition, both “ashoub2015” and “rollins2013” combined drought and heat stress. The meta-analysis “shaar-moshe2015” compared drought related genes from different plant species. Although many of these works (10) sampled leaves, other tissues were also analyzed in some of them (mainly shoots, roots, spikes and grain).

**Table 4:** **Studies from the literature assessing protein or transcript expression changes in response to drought in barley**.

**Alias**	**Publication**	**Type**	**Genotype**	**Stress**	**Develop. Stage**	**Tissue sampled**	**# DE tags**
abebe2010	Abebe et al., 2010	ma	c	d	Grain-filling	Lemma, palea, awn, seed	240
ashoub2013	Ashoub et al., 2013	p	l	d	4-leaves	Leaf	25
ashoub2015	Ashoub et al., 2015	p	w	d, h, c	2 leaves, 4 leaves	Leaf	40
chmielewska2016	Chmielewska et al., 2016	p	c	d	3-leaves	Leaf, root	134
guo2009	Guo et al., 2009	ma	c, w, l	d	Flowering	Leaf (flag)	188
hubner2015	Hübner et al., 2015	r	w	d	Flag leaf emerged	Spikelets	495
kausar2013	Kausar et al., 2013	p	c	d	3-d old seedlings	Shoot	32
matsumoto2014	Matsumoto et al., 2014	ma	c	^*^	4-d old seedlings	Root, shoot	66
rollins2013	Rollins et al., 2013	p	c, l	d, h, c	Heading	Leaf	99
shaar-moshe2015	Shaar-Moshe et al., 2015	me	-	d	-	-	2730
talame2007	Talame et al., 2007	ma	c	d	4-leaves	Leaf	127
vitamvas2015	Vitamvas et al., 2015	p	c	d	2-leaves	Crown	68
wang2015	Wang et al., 2015	p	w, c	d	2-leaves	Leaf	26
wehner2015	Wehner et al., 2015	QTL	c, l	d	BBCH 10, 7 DAS	-	33
wehner2016	Wehner et al., 2016	eQTL	c, l	d	BBCH 10, 7 DAS	Leaf	14
weldelboe2012	Wendelboe-Nelson and Morris, 2012	p	c	d	7 DAS	Leaf, root	69
worch2011	Worch et al., 2011	ma	c, w	d	Post-anthesis	Grain	137

An alias was assigned to each study, to facilitate referring to them. There are different approaches in the comparison dataset, including microarrays (ma), proteomics (p), RNAseq (r), meta-analysis (me), a QTL study and one based on eQTLs. The genotypes used for the experiments involve barley cultivars (c), landraces (l) or wild barley (w). The type of stress applied was drought (d), heat (h), drought and heat combined (c), or dessication, salt and ABA in the case of “matsumoto2014” (^*^). Stresses were applied during different developmental stages, and the tissue sampled was varied also. Finally, the number of differentially expressed tags (transcripts, genes, proteins) included in the comparison dataset is shown (# DE tags).

**Figure 7:**
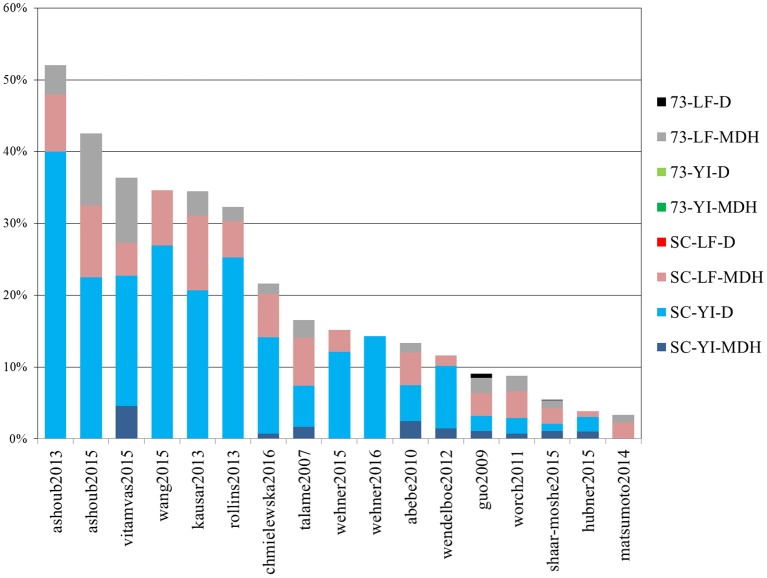
**Percentage of differentially expressed tags from other studies which were identified in the present work**. Bars indicate the percentage of differentially expressed tags (proteins, genes or isoforms) from other studies which were identified in this work. Each color represents the contribution of each contrast. The list of studies used for comparison is given in Table 4.

Out of 4389 DE tags (proteins, genes and transcripts) reported overall in the studies above, more than half (2730) were barley genes included in the meta-analysis “shaar-moshe2015” and, indeed, that study matches the largest number of DE tags of the current work. However, in relative terms, the most similar were those of “ashoub2013,” “ashoub2015,” “vitamvas2015,” “wang2015,” “kausar2013,” and “rollins2013,” in decreasing order, whose DE tags were also found in the present study in proportions ranging from 52 to 32% (Figure 7). Interestingly, these are all proteomics studies. The next most similar study, “chmiewlewska2012,” and also “wendelboe2012,” were both proteomics studies. However, in contrast with the previous ones, focused on leaf tissue, these also included roots. Note that when considering only DE proteins from leaf samples, the agreement with “chmielewska2012” increased approximately from 22% to around 27%. On the other hand, DE transcripts from Scarlett YI under D matched the largest percentage of DE tags from the surveyed studies.

We also recorded the number of DE tags found in individual contrasts of our study, which had already been identified in previous studies. These figures for the four main contrasts of our study, Scarlett YI under D, Scarlett YI under MDH, SBCC073 LF under MDH and Scarlett LF under MDH, were 49, 30, 58, and 55%, respectively. The largest figures found for the leaf contrasts likely reflect the prevalence of studies which sampled LF tissues.

A total 466 DE isoforms were not found in previous studies, whereas 153 were in just one study and 54 in two. Only 23 DE isoforms were in common in three or more studies. These DE isoforms included several 70 and 90 kDa heat shock proteins, a S-methyltransferase and a S-adenosylmethionine synthase 2 from S-adenosyl-L-methionine cycle, an N-methyltransferase involved in choline biosynthesis, transcripts related with photosynthesis and carbon fixation, a sucrose synthase, a phosphoglycerate mutase and a triose-phosphate isomerase, a glutathione peroxidase, a ferredoxin-NADP+ reductase, a phenylalanine/tyrosine ammonia-lyase, a ClpC subunit from an ATP-dependent Clp protease, an ATP synthase and a V-type H+-transporting ATPase subunit, an aspartate kinase, a protein with Potato inhibitor I family domain and a spermidine synthase (Table 5).

**Table 5:** **Differentially expressed isoforms found in three or more previous studies**.

**DE isoform**	**Gene name**	**73-LF-MDH**	**SC-LF-MDH**	**SC-YI-D**	**abebe2010**	**ashoub2013**	**ashoub2015**	**chmielewska2016**	**guo2009**	**hubner2015**	**kausar2013**	**matsumoto2014**	**rollins2013**	**shaar-moshe2015**	**talame2007**	**vitamvas2015**	**wang2015**	**wehner2015**	**wehner2016**	**wendelboe2012**	**worch2011**
00425	5-methyltetrahydropteroyltriglutamate-homocysteine S-methyltransferase																				
01438	heat shock 70kDa protein 1/8																				
30291	photosystem II																				
03771	ribulose bisphosphate carboxylase/oxygenase activase, chloroplastic																				
23857	phosphoethanolamine < i>N- < /i>methyltransferase																				
49313	ribulose-bisphosphate carboxylase																				
15018	heat shock 70kDa protein 1/8																				
19971	ATP synthase alpha/beta family, nucleotide-binding domain																				
46536	sucrose synthase																				
01544	heat shock 70kDa protein 1/8																				
46824	2,3-bisphosphoglycerate-independent phosphoglycerate mutase																				
22980	heat shock protein 90kDa beta																				
03577	glutathione peroxidase																				
43420	V-type H+-transporting ATPase subunit B																				
20214	triose-phosphate isomerase																				
13150	S-adenosylmethionine synthase 2																				
39096	phenylalanine/tyrosine ammonia-lyase																				
02838	ATP-dependent Clp protease ATP-binding subunit ClpC																				
18227	spermidine synthase																				
25293	ferredoxin-NADP <small> <sup>+ </sup> </small> reductase																				
49597	Potato inhibitor I family																				
33995	-unknown-																				
15965	aspartate kinase																				

*Each row corresponds to a differentially expressed (DE) isoform which was observed in three or more previous studies. Fields include annotated gene name of each DE isoform, and the contrast in which it was declared as DE (73: SBCC073, SC: Scarlett, YI: young inflorescences, LF: leaves, D: severe drought treatment, MDH: mild drought and heat treatment). The presence of the DE isoform in a given study is highlighted with gray background*.

### Analysis of co-expressed genes

DE isoforms were clustered based on their expression patterns across samples (Figure S11), with the aim of identifying shared regulatory motifs in their upstream genomic regions. We obtained 23 clusters, 14 of them with more than 10 isoforms (Table S7). Several clusters contained mostly isoforms from a given contrast while others had mixed DE tags from different treatments (Figures S12, S13).

In order to validate the expression-based gene clusters, they were tested for Gene Ontology (GO) enrichment. Moreover, to test the hypothesis that co-expressed genes might share *cis*-regulatory sequences, their upstream sequences were subjected to motif discovery algorithms and the DNA motifs found were annotated. Finally, the resulting regulatory motifs were compared to the binding predictions of DE expressed TFs identified in this work, trying to link these TFs to clusters of DE tags.

**Figure 8:**
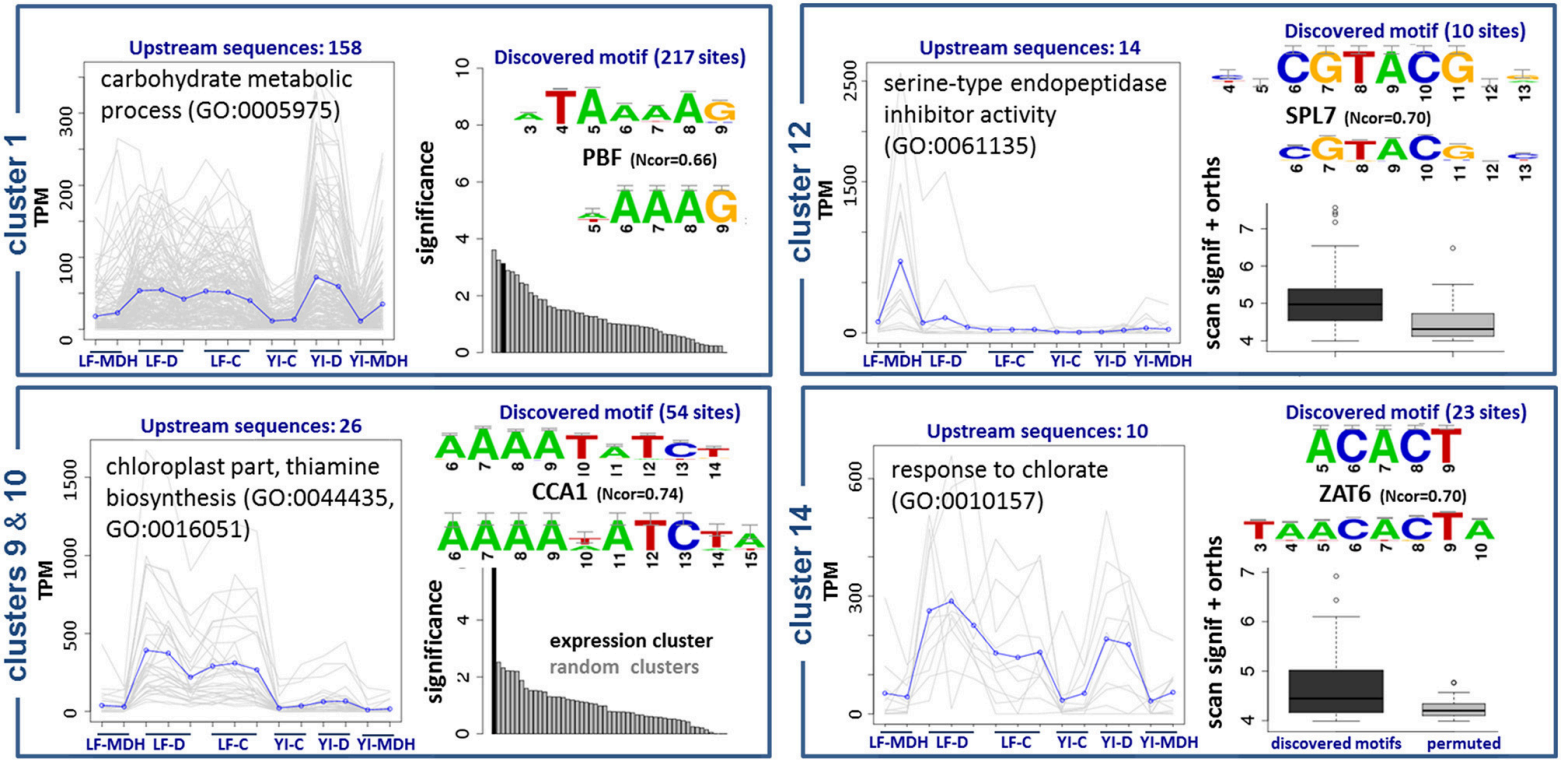
**Enriched DNA motifs in promoters of differentially co-expressed isoforms**. Gene Ontology enrichment and regulatory motifs discovered in 5 clusters of co-expressed isoforms. For each cluster, a plot is shown on the left with the expression profile, where LF and YI correspond to leaf and young inflorescence tissues, and G, D and C to greenhouse, chamber and control replicates, respectively. Regulatory motifs are shown on the right side of each cluster box, with the discovered consensus sequence on top and the most similar motif in footprintDB aligned below. Cluster 10 was found to be very similar to cluster 9, and thus is not shown. The evidence supporting the motifs of clusters 1, 9 and 10 is their significance (black bars) when compared to negative controls (gray bars). Motifs of clusters 12 and 14 (dark boxplots) have higher scores than their shuffled motifs (gray boxplots) when scanned along the cluster upstream sequences and their *Brachypodium distachyon* orthologues.

The results are summarized in Figure 8. Upstream sequences of genes from cluster 1, with functional annotations related to the metabolism of carbohydrates, contain a wtATAAAAGw site, which is similar to motifs of TATA-binding proteins and Dof TFs (Yanagisawa, 2002). We observed a C2C2-Dof TF up-regulated in SBCC073 LF under MDH (see previous sections), although we were not able to identify DNA-binding domains associated to it. Therefore, we cannot confirm whether or not C2C2-Dof protein binds to this motif to regulate genes in cluster 1, but the possibility deserves further investigation. Promoter sequences of genes in clusters 9 and 10, which group mostly transcripts down-regulated in LF under MDH, contain sites identical to the consensus of CCA1/LHY, which belongs to the MYB/SANT family (Green and Tobin, 1999). These sites were independently predicted by oligo-analysis (AAAATATCTy) and dyad-analysis (aAAAkaTCTw), indicating that they are high-confidence predictions. Genes of this cluster are annotated as components of thiamine biosynthesis in the chloroplast. Accordingly, CCA1/LHY, which was up-regulated in SBCC073 and Scarlett samples under MDH, binds to the same motif (aAAATATCTkY). Cluster 12 had predicted yaCGTACGtr *cis*-elements. Genes in this cluster were induced in LF under MDH, and are annotated as heat shock proteins. Finally, genes in cluster 14 are annotated as components of salinity response, and share *cis*-elements of consensus smACACTbm.

Out of 11 DE TFs, 7 were associated with DNA-binding domains (Table 6), including CCA1/LHY (see above), the MYB-related TF of unknown function DE in SBCC073 LF under MDH, the MADS-MIKC up-regulated in Scarlett YI under D (AwRGaAAaww), the B3-ARF TFs induced in Scarlett YI either under D or MDH (yTTGTCtC), the bZIP up-regulated in Scarlett LF under MDH (cayrACACGTgkt) and the AP2/ERF-AP2 down-regulated in SBCC073 LF under MDH (CACrrwTCCCrAkG). It is possible that these genes were in part regulating the changes in gene expression in response to the treatments. However, these could not be linked to the motifs identified in promoters.

**Table 6:** **Predicted DNA motifs for differentially expressed transcription factors**.

**Isoform**	**Pfam**	**Contrast**	**Up/Down-regulated**	***E*-value**	**DNA motif**	**SwissProt**
comp690102_c3	AP2/ERF-AP2	73-LF-MDH	dn	7.00E-79	CACrrwTCCCrAkG	Q8LSN2-BnBBM2
comp700847_c0	B3-ARF	SC-YI-D	up	7.00E-150	yTTGTCtC	Q6YZW0-OsARF21
comp61422_c0	B3-ARF	SC-YI-MDH	up	1.00E-98	yTTGTCtC	Q85983-OsARF11
comp59053_c0	bZIP	SC-LF-MDH	up	7.00E-42	cayrACACGTgkt	–
comp688195_c0	C2C2-Dof	73-LF-MDH	up	–	–	Q93ZL5-AtCDF2
comp67310_c0	CCA1/LHY	SC-YI/LF-MDH	up	0.00E+00	waGATAttt	Q6R0H1-AtLHY
comp53438_c1	CCA1/LHY	73-YI/LF-MDH	up	0.00E+00	waGATAttt	Q6R0H1-AtLHY
comp51250_c2	MYB-related	73-LF-MDH	up	5.00E-46	waGATwttww	–
comp61039_c0	MADS-MIKC	SC-YI-D	up	8.00E-61	AwRGaAAaww	Q6EU39-OsMADS6
comp689206_c7	MYB-related	73-LF-MDH	up	–	–	B3H4X8-AtTCL2
comp66417_c0	MYB-related	SC-LF-MDH	up	–	–	B3H4X8-AtTCL2
comp64196_c0	TIFY	SC-LF-MDH	up	–	–	Q6ES51-OsTIFY6B
comp702448_c0	TUB	SC-LF-MDH	dn	–	–	Q7XSV4-OsTULP7

*DE isoforms which were annotated as TFs in all the contrasts (73: SBCC073, SC: Scarlett, YI: young inflorescences, LF: leaves, D: severe drought treatment, MDH: mild drought and heat treatment) are shown along with their iTAK-annotated Pfam domains, whether they were induced (up) or repressed (dn), the BLASTP E-value of homologous TFs, the sequence motif predicted by footprintDB and the best SwissProt hit, along with its gene name prefixed with acronym of the organism (At: Arabidopsis thaliana; Bn: Brassica napus; Os: Oryza sativa subsp. japonica)*.

## Discussion

In this work, ***de novo* assemblies** of Spanish landrace SBCC073 and elite cultivar Scarlett were generated. These assemblies had a larger number of isoforms and genes than current barley references. This could be an effect of sequencing errors and non-coding sequences being expressed, but also of absence of actual transcripts from the references. Nonetheless, the use of all available reference sequences (Morex, Barke, Bowman, Haruna Nijo) led to the confirmation of a substantial percentage of those isoforms, allowing the identification of more assembled isoforms than using any of them separately. This highlights the variability in gene content between genome references, which poses a problem when working with non-reference genotypes as in the present study. In light of this, an advantage of *de novo* assemblies resides in recovering genotype-specific transcripts and in reducing mapping errors produced by polymorphisms. Therefore, using them as reference, as we have done in this study, allows diminishing the proportion of unmapped reads and increasing mapping accuracy, which is essential for gene expression assays. Moreover, we tested three different pipelines for differential expression, and those based on *de novo* assemblies had a better agreement with RT-qPCR results.

As *de novo* assembled sequences are by definition transcript isoforms, it is natural that downstream analyses are performed with them instead of genes. In this context, a gene is in fact a cluster of isoforms. In order to work with them some compromises have to be taken. For instance, choosing a representative sequence for the gene, frequently the longest, might lead to losing useful information from splicing/assembly variants. Moreover, working with isoforms allowed us to go a step further, and discriminate the different isoforms which appeared in response to different stresses, in the tissues of both barley genotypes. For instance, we identified several genes with multiple DE isoforms, which are candidates for future studies about their role, and regulation of their expression, in response to stress. Nevertheless, we carried out assembly and gene expression analyses both with isoforms and genes, which confirmed that the results are robust in general terms, but also that there are differences. Working with isoforms has also drawbacks (Conesa et al., 2016). Isoforms which result from *de novo* assembly are not always complete transcripts but, rather, fragmented sequences or, in the worst cases, chimeric. Moreover, groups of isoforms may bear both transcripts from a single gene, but also paralogues, or expressed pseudogenes, which are closely related to the former. This can also happen in the opposite direction, if isoforms from a single gene are eventually split into different genes. Nonetheless, our study suggests that the results obtained with isoforms are more accurate, as seen in the agreement of results from different pipelines, RT-qPCR results, and with proteomics studies.

Plants from Scarlett and SBCC073 were subjected to severe drought and mild drought combined with heat, during the reproductive stage, and **physiological responses** were measured. Water-stressed plants showed reduced daily loss of water, increased absolute leaf water potential, changes in stomatal conductance, and reduced tiller number at the end of the experiment. There were also differences in response to stress shown by the two genotypes, indicating different strategies of adaptation to stress. Absolute leaf water potential under severe drought was higher in Scarlett than in SBCC073, indicating worse hydric status in Scarlett. Moreover, under combined mild drought and heat, Scarlett exhibited the lowest tiller number, with relative water content comparable to plants under severe drought. In comparison, both measurements were close to that of well-watered plants in SBCC073, under the combined stress. Taken together, these results indicate that Scarlett was more susceptible to mild drought and heat than SBCC073. Experiments carried out in pots, like this, have the disadvantage of not mimicking natural conditions perfectly. On the other hand, experiments in controlled settings actually help to limit variation due to interaction with environment. For instance, rooting depth is kept out of the equation as, although the pots were large, the roots readily explored all soil volume. Hence, potential genotypic differences in soil exploring capacity cannot be held responsible for the genotypic disparities in physiological measurements. Given that soil conditions and water availability were similar for the two genotypes, it can be concluded that SBCC073 was more drought tolerant than Scarlett. Also, growth differences between treatments, carried out in the growth chamber and in the greenhouse, could have been affected by different day lengths, long and constant in the first, and still long but decreasing in the greenhouse.

Regarding **gene expression**, the responses to the stresses were specific of each tissue and genotype. Drought almost did not impact SBCC073, whereas the combination of mild drought and heat only affected its leaves. In contrast, gene expression in both Scarlett tissues was strongly altered in the greenhouse, whereas severe drought alone impacted young inflorescences only. The coupled physiological and transcriptional responses to abiotic stresses are among the main findings of this study.

Overall, we found few changes in **leaves** under severe drought stress. Although related studies found more differences in gene expression in leaves, most of them studied early responses and only a few addressed prolonged stresses, as in the present study. Processes involved in plant responses to water deficit are different depending on the temporal scale, being those related with drought resistance and grain production, like phenology adjustment, acclimation, fertility and harvest index, affected by medium- to long-term water scarcity (Passioura, 2004). Severe brief stresses, which are rare in the field, are more related with plant survival (Passioura, 2002). Nonetheless, another study focused on long-lasting water and heat stress (Ashoub et al., 2015) reported many gene expression changes. However, that study involved wild barley seedlings starting at the stage of two leaves, quite different from the conditions of the present study. Leaves from adult plants, like the ones in our study, are expected to show different responses to drought than those of seedlings (Blum, 2005). Mature flowering plants could have a more limited transcriptional response to prolonged drought stress due to acclimation or enhanced tolerance, which could be achieved, for example, through selective senescence of older leaves or the development of a deep root system (Blum, 2005, 2009). Studies similar to ours, in which the stress conditions were maintained for a long period, and samples were taken from adult plants, have provided contrasting results. The closest result to ours was found by Rollins et al. (2013), who reported no changes in leaf proteome of mature barley plants under drought stress, but apparent changes due to heat. Also, Chmielewska et al. (2016) identified few proteomic changes in leaves, but greater changes in roots, in a drought tolerant genotype in comparison with a susceptible one, although this study surveyed seedlings exposed to a short stress period. We cannot rule out that our tolerant genotype, SBCC073, shows a similar response in roots. More generally, sampling a single tissue underestimates the consequences of stress in the whole plant, as seen in our study in Scarlett young inflorescences under severe drought stress. Others, did find differentially expressed genes in flag leaves of adult barley plants (Guo et al., 2009) or changes in protein expression in mature leaves of wheat drought tolerant genotypes (Ford et al., 2011).

In contrast with the drought treatment, we found numerous differentially expressed transcripts in leaves under combined drought and heat stress. There is scarce information about the optimum temperature for barley growth. We can assume that it is close to the one reported for wheat, whose optimum range is between 18° and 23°C (Slafer and Rawson, 1995; Porter and Gawith, 1999). A previous study showed that high temperature (25°C) resulted in rapid progression through reproductive development in long days (Hemming et al., 2012). The temperatures in the greenhouse clearly exceeded that range for most of the experimental period and, therefore, these plants experienced a combination of heat and drought stress. These conditions are expected to occur more frequently in many regions according to future climate conditions expectations. However, we have to be aware that other environmental factors could be affecting plants in the greenhouse, such as a mild powdery mildew infection, presence of phytophagous insects, and variable natural photoperiod.

In such conditions, there were several DE isoforms in common in both genotypes. For example, transcription of CCA1/LHY was induced in Scarlett and SBCC073, in both leaves and young inflorescences. The observed changes in expression of CCA1/LHY might be related to photoperiod rather than to tolerance to stress, given that CCA1/LHY is a component of the circadian clock (Campoli et al., 2012; Deng et al., 2015), and other genes related with circadian clock were also differentially expressed in leaves under mild drought and heat, like HvPRR1/TOC1 (Campoli et al., 2012) and an homolog of Arabidopsis adagio-like protein 3. Even so, CCA1/LHY has been shown to be controlled by heat in plants (Karayekov et al., 2013), including barley (Ford et al., 2016), and by other abiotic stresses (Grundy et al., 2015), including osmotic stress (Habte et al., 2014). Also, among differentially expressed transcripts in leaves, the most recurrent were those related with polyamines (like spermine and spermidine), which were identified in leaves from both genotypes, under severe drought alone and under drought combined with heat. These are small aliphatic amines which have been associated to numerous stresses in plants, including osmotic stress and heat (Bouchereau et al., 1999), and their knock-out mutants in Arabidopsis show increased susceptibility to drought stress (Yamaguchi et al., 2007). However, their specific roles in drought stressed plants remain obscure (Capell et al., 2004; Do et al., 2013).

Besides that, Scarlett leaves displayed more numerous and functionally diverse differentially expressed transcripts than SBCC073, under mild drought and heat. Despite presenting comparable stomatal conductance to SBCC073, Scarlett showed increased responses in genes related to photosynthesis and carbon fixation metabolism, as well as antioxidant enzymes. Also, this genotype seems to react more actively to pathogen attack under MDH, as seen by the increased biosynthesis of molecules related to defense responses. Another interesting genotypic difference was that glycine betaine biosynthesis was induced in SBCC073, whereas in Scarlett trehalose biosynthesis was induced instead. These two compounds have an alleged osmoprotectant function in organisms. While glycine-betaine is well known in plants, including cereals (Ashraf and Foolad, 2007), trehalose is not common in plants (Majumder et al., 2009). These results point toward the presence of effects on different pathways, and different genotypic strategies to cope with the combination of stresses encountered in the greenhouse treatment.

In **young inflorescences**, there were noticeable changes in gene expression in Scarlett, but almost none in SBCC073, in both stress treatments. As in leaves, this could indicate that Scarlett inflorescences were suffering more from stress than those of SBCC073. A similar interpretation was made by Hübner et al. (2015), who found a larger proportion of differentially expressed genes for this plant organ in response to stress in sensitive genotypes of wild barley. It is intriguing that inflorescences from Scarlett in the greenhouse showed primarily repressed transcripts, most of them related with metabolism of carbohydrates, reorganization of cell wall and biosynthesis of secondary metabolites. Also, two transcripts involved in indole-3-acetic acid (IAA) biosynthesis were repressed: an L-tryptophan transaminase, which catalyzes the conversion of tryptophan to indole-3-pyruvate, and an indole-3-pyruvate monooxygenase, which yields IAA. This is a key auxin, a phytohormone which regulates many critical developmental processes (Woodward and Bartel, 2005). Barley developing inflorescences are a source of IAA (Wolbang et al., 2004), involved in modulation of stem growth and of floret primordia development (Leopold and Thimann, 1949). We could speculate that this could be an attempt to delay spike development in the face of severe stress.

Differentially expressed transcripts were compared with those from **related studies**. Disparities with other studies partly reflect differences in experimental set up and vegetal material assessed, but other causes are also possible. Interestingly, agreement was better with works based on proteomics than on transcriptomics. This may reflect a statistical bias, due to the choice of strict significance thresholds in our case and in proteomics studies. In fact, the number of differentially abundant proteins reported from proteomics studies was low, which could explain in part the large percentage of coincidences. On the other hand, RNAseq sampling and expression range is different from that of microarrays (Ozturk et al., 2002), which predominated in the gene expression datasets used for comparison, which could favor obtaining results closer to those of proteomics. Also, the fact that our analysis focused on isoforms, instead of on genes (actually, clusters of isoforms, obtained from *de novo* assembly), could have contributed to this. There was only one study using RNAseq in the comparison dataset (Hübner et al., 2015), but similarities with it were also scarce. These authors sequenced transcripts from barley immature spikelets subjected to prolonged water stress, which is rather similar to our experiment. However, they worked with wild barley, whereas this study employed a landrace and an elite cultivar. Wild barley holds much more diversity than cultivated types, with considerable variation in physiological and phenotypic characteristics, and presents specific environmental adaptations to stress like temperature and rainfall (Ellis et al., 2000; Hübner et al., 2013). Therefore, it is feasible that the responses to abiotic stresses of wild barley are different to those of cultivated genotypes. In addition, the methodology in that study, an approach based on RGA, was also different from the one adopted here. As mentioned above, we show that such method produced different outcomes than *de novo* assemblies.

Overall agreement between studies was limited, as seen by the few DE isoforms found in common in three or more studies. A previous meta-analysis of gene expression in response to drought (Shaar-Moshe et al., 2015) also detected few common differentially expressed transcripts between studies, although in this case the comparison involved different plant families. This notwithstanding, some processes are recurrently found in drought studies in barley, including ours, independently of the diversity of genotypes and environmental conditions employed. Hence, these could play central roles in the response of barley to abiotic stress. Many of these have already been discussed and reviewed, like the role of polyamines (see above) (Guo et al., 2009; Abebe et al., 2010; Ashoub et al., 2013), proteases (Ford et al., 2011; Ashoub et al., 2013), glycine betaine and other osmoprotectants (Abebe et al., 2010; Ashoub et al., 2013, 2015; Chmielewska et al., 2016), ascorbic acid (Guo et al., 2009; Wendelboe-Nelson and Morris, 2012; Wang et al., 2015; Chmielewska et al., 2016), lipoxygenases (Wendelboe-Nelson and Morris, 2012; Ashoub et al., 2015), aldehyde dehydrogenase (Guo et al., 2009), and also components of photosystem II, carbohydrates metabolism, heat shock proteins, methionine metabolism, or antioxidant enzymes like catalases, which are well known to be involved in stress responses in plants (Krasensky and Jonak, 2012; Marco et al., 2015).

In order to understand the role of differentially expressed genes, it is important to analyze how these genes are orchestrated. Here, this was accomplished by discovering potential *cis*-elements within upstream promoter sequences. Indeed, this study shows that RNAseq can be exploited to obtain biologically relevant conclusions from **co-expressed genes** using currently available barley genomic resources. As a proof of concept, the CCA1/LHY TF, up-regulated in leaves under mild drought and heat, was associated to two clusters of repressed transcripts, which harbor high-confidence CCA1 binding sites in their promoter sequences. Genes in those clusters were related to thiamine biosynthesis in the chloroplast, an early response to stress known to be linked to the circadian clock (Bocobza et al., 2013; Wang et al., 2016). Transcripts from thiamine biosynthesis were repressed in another study assessing barley under drought (Talame et al., 2007), indicating that thiamine could play an important role in drought response, maybe regulating function of enzymes for which it is a cofactor, enhancing tolerance to oxidative damage, or as a signaling molecule in adaptation mechanisms to abiotic stress (Tunc-Ozdemir et al., 2009; Goyer, 2010). Therefore, we were able to associate gene regulation apparently elicited by CCA1/LHY with a previously known stress response linked to regulation of thiamine biosynthesis, through analysis of DNA-binding motifs.

Besides CCA1/LHY, we were able to identify other promoters and DNA-binding affinities of TFs. A motif involved in the regulation of heat shock proteins matches a SBP zinc-finger protein SPL7, which has been described as a TF related to heat stress in rice (Yamanouchi et al., 2002). Genes from another cluster shared a motif whose best hits were Arabidopsis ZAT6, belonging to a family of zinc-finger repressors involved in responses to salt stress (Ciftci-Yilmaz et al., 2007), and AZF2, a C2/H2 zinc-finger which negatively regulates abscisic acid-repressive and auxin-inducible genes under abiotic stress conditions (Kodaira et al., 2011). Moreover, among hundreds of differentially expressed transcripts, only 11 TFs were found in this study (including CCA1/LHY). As an example, we found differential expression of transcripts of a MYB-related protein, whose closest SwissProt homologs are single-repeat R3 MYB TFs from Arabidopsis. These are involved in epidermal cell fate specification, more specifically in regulation of trichome development (Gan et al., 2011). Therefore, this MYB-related protein could have a similar role of that of GT factors in wheat, which have been related to drought tolerance and trichome development (Zheng et al., 2016). Some of the TFs identified here have already been associated with abiotic stress in rice or Arabidopsis. In example, we found a bZIP TF whose DNA-binding motif corresponds to that of ABRE (ABA-responsive element) *cis*-element, and thus could be regulating ABA-responsive genes (Nakashima et al., 2014). We also found an AP2/ERF-AP2 TF differentially expressed in SBCC073 leaves. The AP2/ERF is a large family of plant-specific TFs, which includes dehydration-responsive element-binding (DREB) proteins, involved in the activation of drought responsive genes (Mizoi et al., 2012). However, the TF reported here was similar to BABY BOOM genes from *Brassica napus*, in which they promote embryo development (Boutilier et al., 2002). We also found differentially expressed transcripts related to a MADS-MIKC homolog of OsMADS6, related with floral organ and meristem identities in rice (Li et al., 2010), up-regulated in Scarlett developing inflorescences under drought; an uncharacterized MYB-related TF, in SBCC073 leaves only; a C2C2-Dof, similar to Arabidopsis CDF2, which regulates miRNAs involved in control of flowering time (Sun et al., 2015); a TF of the TIFY family, whose members are responsive jasmonic acid and to abiotic stresses (Ye et al., 2009); a TUBBY-like protein (TULP), which have been associated to sensitivity to ABA in Arabidopsis (Lai et al., 2004); and two transcripts matching different B3-ARF (auxin responding factor with B3 domains) from Arabidopsis. Therefore, the responses observed here seem to have only partial overlap with those already described in other plants. For example, NAC TFs (Nakashima et al., 2012) have not been found in this study. Taking advantage of DNA-binding motifs allows linking TFs and groups of co-expressed genes through their common interface, and provides an additional layer of insight on the dynamics of stress responses in plants. Signaling pathways in response to drought in barley, especially depending on type of stress, development stage, tissue and genotype, remain to be deciphered (Gürel et al., 2016), although it is expected that different responses and strategies will be favored in different agronomic contexts.

Well-adapted accession SBCC073 is currently being tested under water stress field conditions in two mapping populations derived from crosses, to search for QTL that control agronomic and physiological traits related to good performance under water stress. The catalog of sequence transcripts and expression profiles from the current study will complement this population-based approach to unravel the genetic control of drought responses which impact grain yield.

## Data accessibility

Raw reads of barley landrace SBCC073 and cultivar Scarlett have been deposited at ENA (study PRJEB12540). Assembled transcripts of SBCC073 and Scarlett are available upon request.

## Author contributions

EI, MPG, and AC, conceived the experiment and designed the greenhouse and growth chamber experiment. CC, AC, and BC designed the sequencing experiments. CC and AC grew and dissected the plants, made physiological measurements and extracted RNA. CC, MJG, and BC analyzed RNAseq data. CC and AC performed RT-qPCR experiments. All authors read and approved the final manuscript.

## Funding

This work was funded by DGA - Obra Social La Caixa [grant number GA-LC-059-2011] and by the Spanish Ministry of Economy and Competitiveness [projects AGL2010-21929, RFP-2012-00015-00-00 AGL2013-48756-R and AGL2016-80967-R]. Carlos P. Cantalapiedra is funded by [grant BES-2011-045905 linked to project AGL2010-21929].

### Conflict of interest statement

The authors declare that the research was conducted in the absence of any commercial or financial relationships that could be construed as a potential conflict of interest.
