# Supplementary material for: Large Differences in Gene Expression Responses to Drought and Heat Stress between Elite Barley Cultivar Scarlett and a Spanish Landrace
Source: Front Plant Sci. 2017 May 1;8:647. doi: 10.3389/fpls.2017.00647 (PMC5410667; doi:10.3389/fpls.2017.00647)
Supplement: Supplementary file 1 [file DataSheet1.PDF]

## *Supplementary Material*

# Large Differences in Gene Expression between Elite Barley Cultivar Scarlett and a Spanish Landrace under Drought and Heat Stress

**Carlos P Cantalapiedra, María J García-Pereira, M Pilar Gracia, Ernesto Igartua, Ana M Casas and Bruno Contreras-Moreira***

*** Correspondence:** Bruno Contreras-Moreira: bcontreras@eead.csic.es

## Supplementary Figures

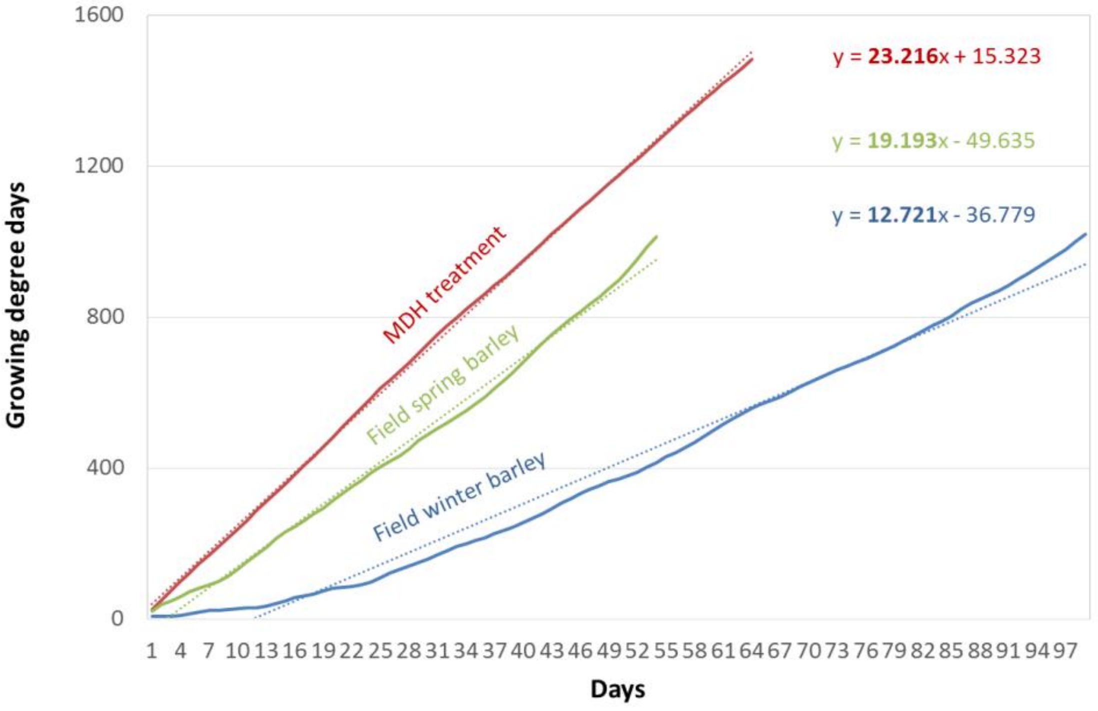


**Figure S1. Growing degree days (GDD) accumulation.**

GDD accumulation in the mild drought and heat (MDH) treatment in this study (red line), and in the same year in 2 locations, Zaragoza (blue line), representing winter barley growing area in the Mediterranean region, and Vienna (purple line), representing spring barley growing area.

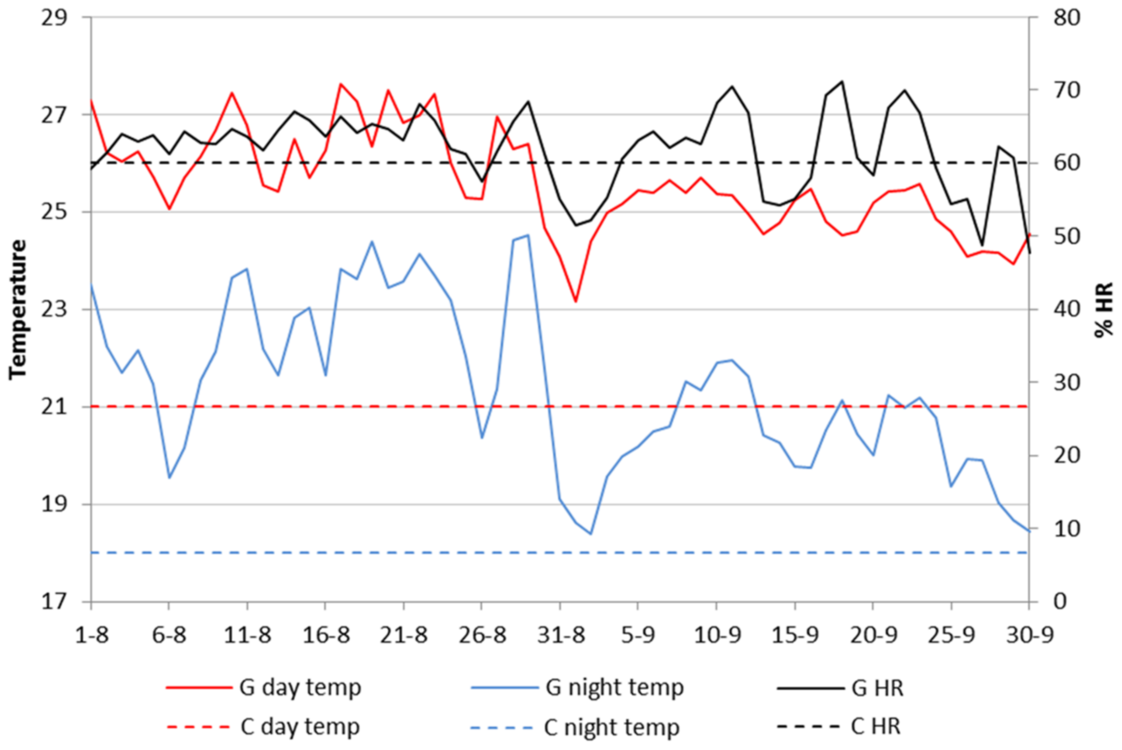


**Figure S2. Relative humidity (% HR) and temperature records during the experiments.**

Greenhouse (G, solid lines) and growth chamber (C, dashed lines) daytime (red lines, left axis) and night temperatures (blue lines, left axis), and relative humidity (black lines, right axis), during the whole experiment, are shown. Daytime and night temperatures were obtained following sunrise and sunset times as of http://www.fomento.gob.es/salidapuestasol/2012/Zaragoza-2012.txt

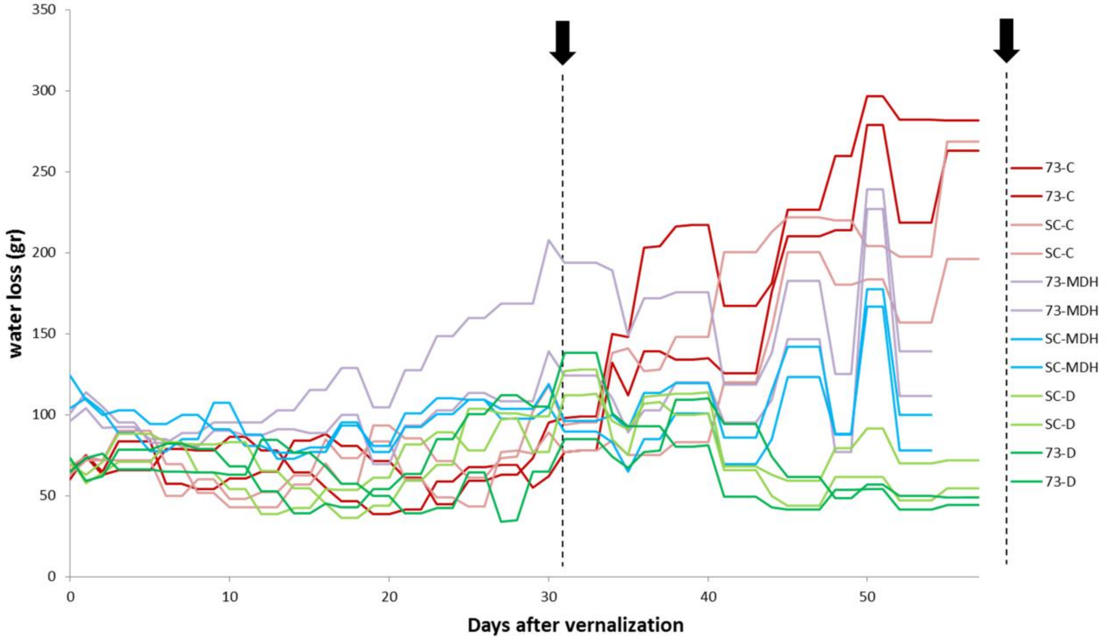


**Figure S3. Average daily loss of water of plants.**

Each series show the average daily loss of water of either SBCC073 (73) or Scarlett (SC) plants, under each of the three treatments: controls (C, red lines), watered to 70% FC; severe drought (D, green lines), 20% FC; and mild drought (50% FC) and heat (MDH, violet and blue lines). Black arrows indicate the time point (days after vernalization) in which treatments were started (left arrow), and the time point in which physiological measures and tissue samples were obtained, after 30 days of treatment (right arrow).

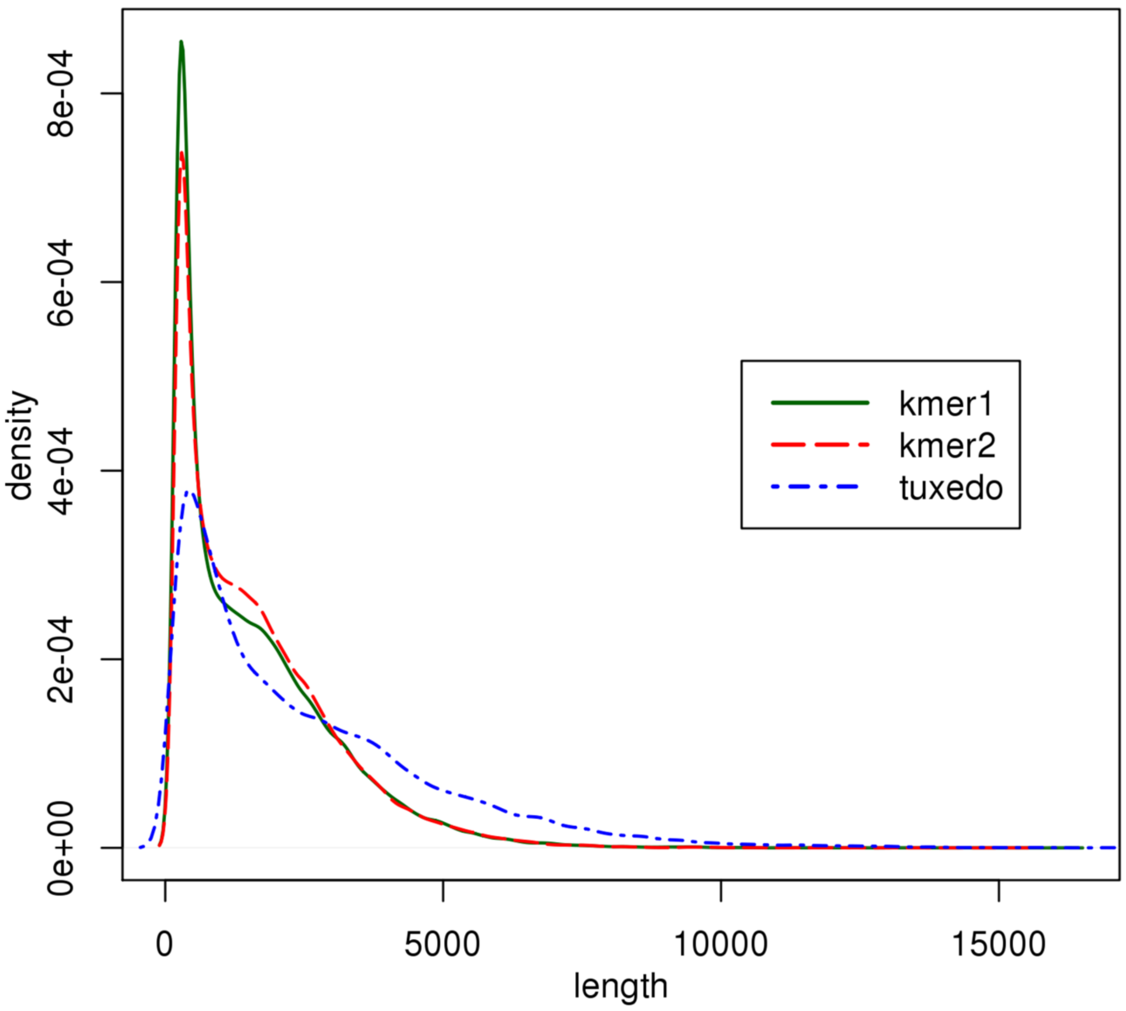


**Figure S4. Distribution of contig lengths obtained from different assemblies.**

Kernel density distribution of contig lengths obtained from Trinity assembly of SBCC073 reads, obtained with '–min-kmer-cov 1' ("kmer1" series) and '—min-kmer-cov 2' ("kmer2" series), and from Reference-Guided Assembly (RGA) with Cufflinks ("tuxedo" series). Number of subcomponents from the stringent assemblies (--min-kmer-cov 2) were closer to those annotated in the reference (78,380 for SBCC073 and 85,785 for Scarlett) than those obtained with –min-kmar-cov 1. Nonetheless, only the first assemblies were used for analysis of differential expression, since the reduction of the mapping space could have an impact on read mapping and subsequent analyses. Moreover, most subcomponents which were not assembled with the more stringent parameters had a single isoform, since the ratio of reduction of subcomponents and transcripts was close to 1 and the average proportion of isoforms per subcomponent increased. Although the relative number of small contigs decreased in the stringent assemblies, both mean contig length and N50 remained almost unchanged, suggesting that some large chimeras could have been split also. On the other hand, the RGA produced 75,204 loci with an average of 1.95 isoforms each. Length of contigs was larger in this assembly than in de novo transcriptomes, with an increase of 1.6-fold in both mean contig length and N50.

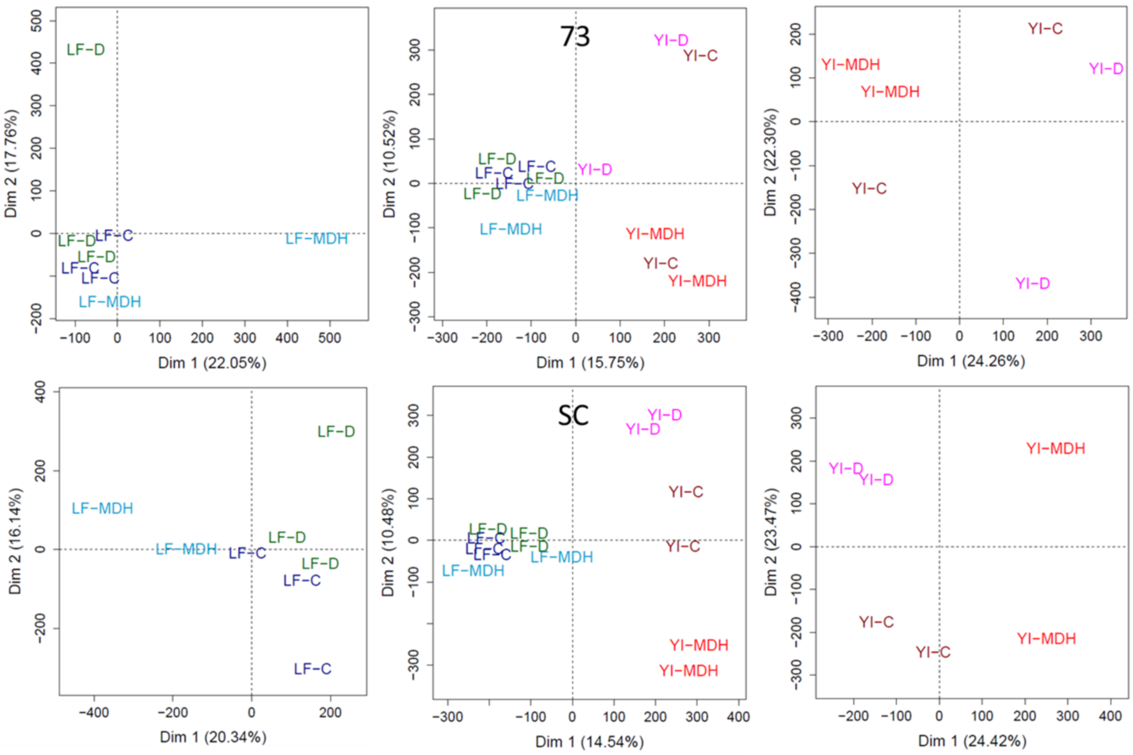


**Figure S5. Principal component analysis of expression estimates.**

Expression estimates in TPM (transcript per million) were obtained from kallisto results. Upper plots: SBCC073 samples (73). Lower plots: Scarlett samples (SC). Left: leaves samples only (LF). Center: all samples. Right: young inflorescences samples only (YI).

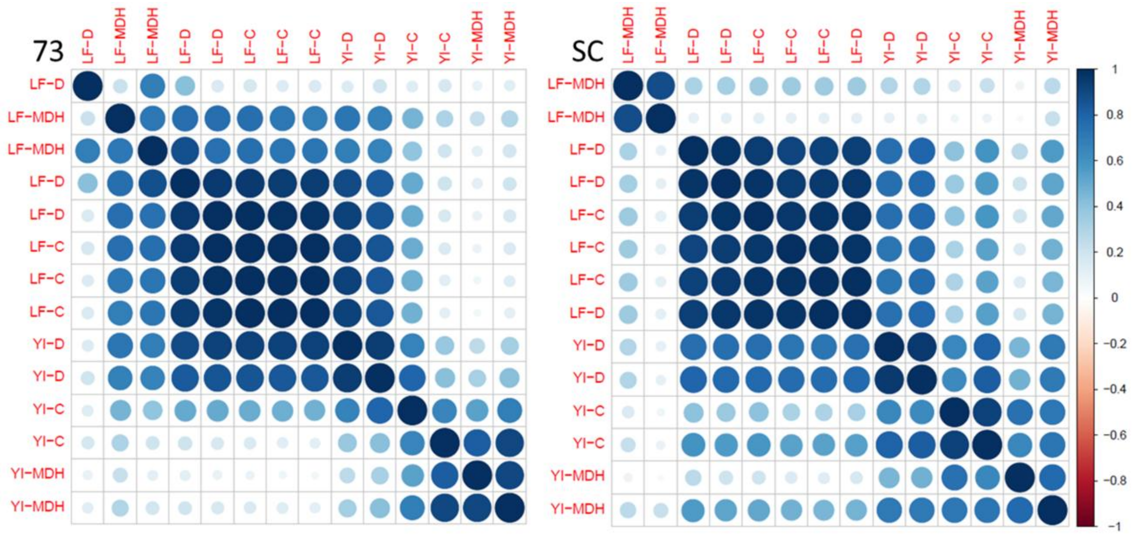


**Figure S6. Correlation plots of expression estimates.**

Expression estimates in TMP (transcript per million) were obtained from kallisto results. Circles are darker and wider as correlation between each two samples increases. LF (leaves) and YI (young inflorescences), from three treatments (D: drought, C: control, MDH: mild drought and heat). Left plot: data for SBCC073 samples (73). Right plot: data for Scarlett samples (SC).

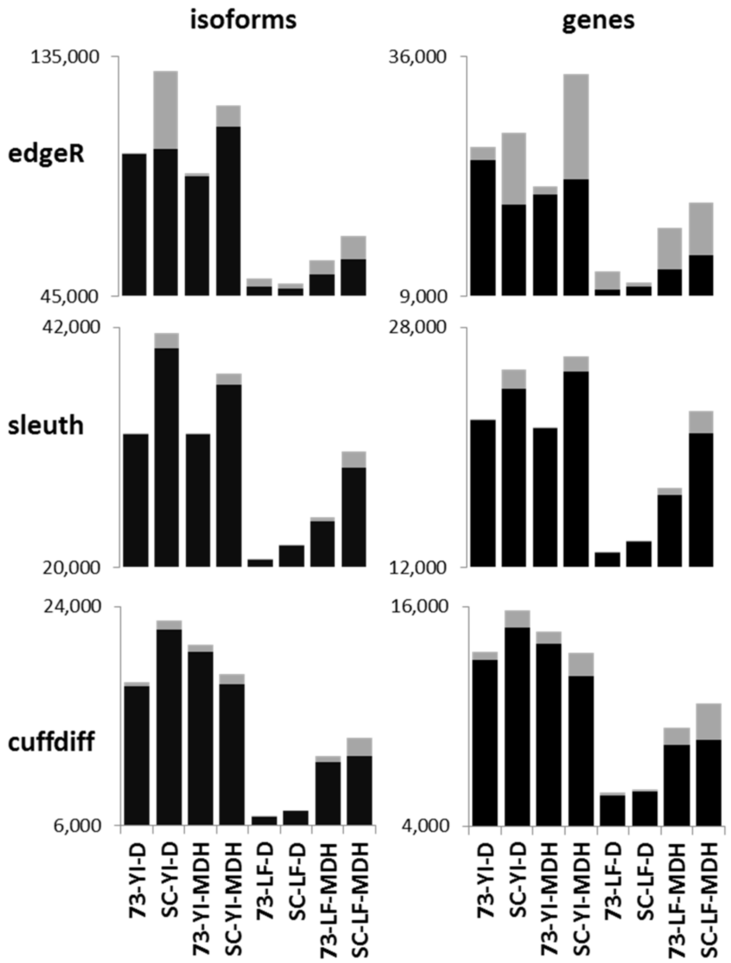


**Figure S7. Cumulative logFC obtained with three pipelines used to test differential expression.**

Each plot shows the logFC for DE (differentially expressed) and non-DE (logFC of DE tags in gray on top of logFC of non-DE tags in black). Plots to the left show results for genes whereas plots to the right show results for isoforms. Results correspond to each contrast for genotypes SBCC073 (73) and Scarlett (SC), involving young inflorescences (YI) or leaves (LF), under drought (D) or mild drought and heat (MDH); as obtained with the three software pipelines: RSEM-edgeR (top), kallisto-sleuth (middle) and Cuffquant-Cuffdiff (bottom). After testing for DE tags, all methods showed similar trends in overall expression response. Of the three methods, RSEM-edgeR was the most sensitive and Cuffquant-Cuffdiff the least one.

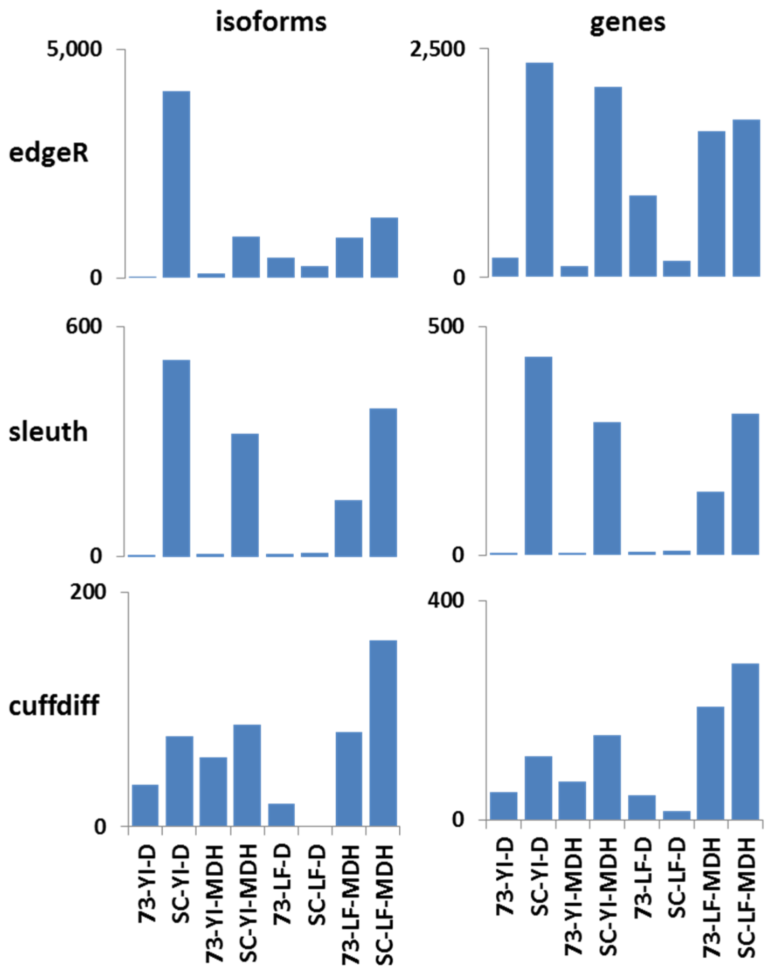


**Figure S8. Number of differentially expressed tags obtained with three pipelines used to test differential expression.**

Number of DE (differentially expressed) genes (left) and isoforms (right) in each contrast for genotypes SBCC073 (73) and Scarlett (SC), involving young inflorescences (YI) or leaves (LF), under drought (D) or mild drought and heat (MDH), as obtained with the three software pipelines: RSEM-edgeR (top), kallisto-sleuth (middle) and Cuffquant-Cuffdiff (bottom). After testing for DE tags, all methods showed similar trends in relative number of genes and isoforms declared as DE. Of the three methods, RSEM-edgeR was the most sensitive and Cuffquant-Cuffdiff the least one, especially for isoforms.

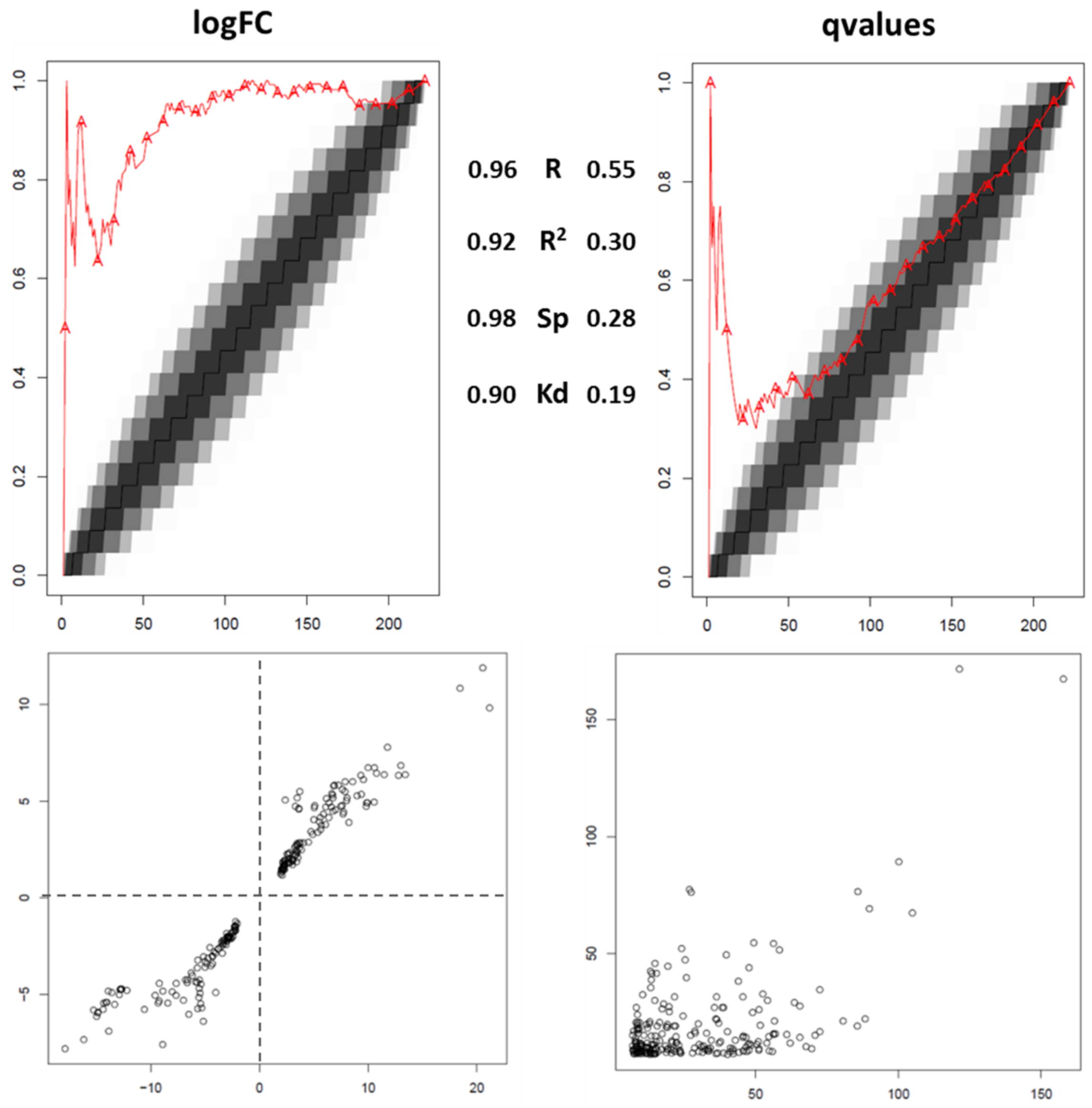


**Figure S9. Comparison of edgeR and sleuth results.**

We compared the agreement of the three methods used to test differential expression. Top: correspondence-at-the-top (CAT) plots and statistics (R: Pearson correlation; Sp: Spearman correlation; Kd: Kendall correlation). Data from contrast SC-LF-MDH (leaves from Scarlett plants under mild drought and heat stress) is shown (red line), whereas gray colors show the average and confidence intervals (0.99, 095, 0.90 and 0.75; darker to lighter) for a hypergeometric distribution. Bottom: scartterplots of actual values obtained from edgeR (horizontal axis) and sleuth (vertical axis). Left: data for logFC; right: data for log(q-value). Dashed line in logFC scatterplot is used to separate positive and negative values. Rank correlation comparing logFC of DE (differentially expressed) isoforms identified by both edgeR and sleuth was good (Sp 0.98), whereas rank based on

FDR adjusted p-values was good only for the most significant DE isoforms. DE genes showed the same trend, although rank correlation was poorer (data not shown). Agreement of either edgeR or sleuth DE sequences with Cuffdiff data was lower (data not shown). CAT plots (Irizarry et al. 2005 Nature Methods 2:345-350) were generated with the R package matchbox (Ross et al. 2011 Prostate 71:1568-1577).

**Genes**

| | 73-YI-D | SC-YI-D | 73-YI-MDH | SC-YI-MDH | 73-LF-D | SC-LF-D | 73-LF-MDH | SC-LF-MDH |
|---|---|---|---|---|---|---|---|---|
| 73-YI-D | - | 0 | 0 | 2 | 0 | 0 | 0 | 0 |
| SC-YI-D | 0 | - | 0 | 15 | 0 | 0 | 2 | 5 |
| 73-YI-MDH | 0 | 0 | - | 2 | 0 | 0 | 2 | 1 |
| SC-YI-MDH | 2 | 15 | 2 | - | 1 | 0 | 1 | 6 |
| 73-LF-D | 0 | 0 | 0 | 1 | - | 0 | 1 | 0 |
| SC-LF-D | 0 | 0 | 0 | 0 | 0 | - | 0 | 2 |
| 73-LF-MDH | 0 | 2 | 2 | 1 | 1 | 0 | - | 31 |
| SC-LF-MDH | 0 | 5 | 1 | 6 | 0 | 2 | 31 | - |

**Isoforms**

| | 73-YI-D | SC-YI-D | 73-YI-MDH | SC-YI-MDH | 73-LF-D | SC-LF-D | 73-LF-MDH | SC-LF-MDH |
|---|---|---|---|---|---|---|---|---|
| 73-YI-D | - | 0 | 0 | 2 | 0 | 0 | 0 | 0 |
| SC-YI-D | 0 | - | 0 | 5 | 0 | 0 | 1 | 4 |
| 73-YI-MDH | 0 | 0 | - | 1 | 0 | 0 | 2 | 0 |
| SC-YI-MDH | 2 | 5 | 1 | - | 0 | 0 | 1 | 2 |
| 73-LF-D | 0 | 0 | 0 | 0 | - | 0 | 0 | 0 |
| SC-LF-D | 0 | 0 | 0 | 0 | 0 | - | 0 | 1 |
| 73-LF-MDH | 0 | 1 | 2 | 1 | 0 | 0 | - | 23 |
| SC-LF-MDH | 0 | 4 | 0 | 2 | 0 | 1 | 23 | - |

**Figure S10. Number of common differentially expressed tags between contrasts.**

Top: common DE (differentially expressed) genes between each two contrasts for genotypes SBCC073 (73) and Scarlett (SC), involving young inflorescences (YI) or leaves ("LF"), or plants under drought (D) or mild drought and heat (MDH). Bottom: common DE isoforms between each two contrasts. Grey background gets darker as the number of DE tags in the intersection increases.

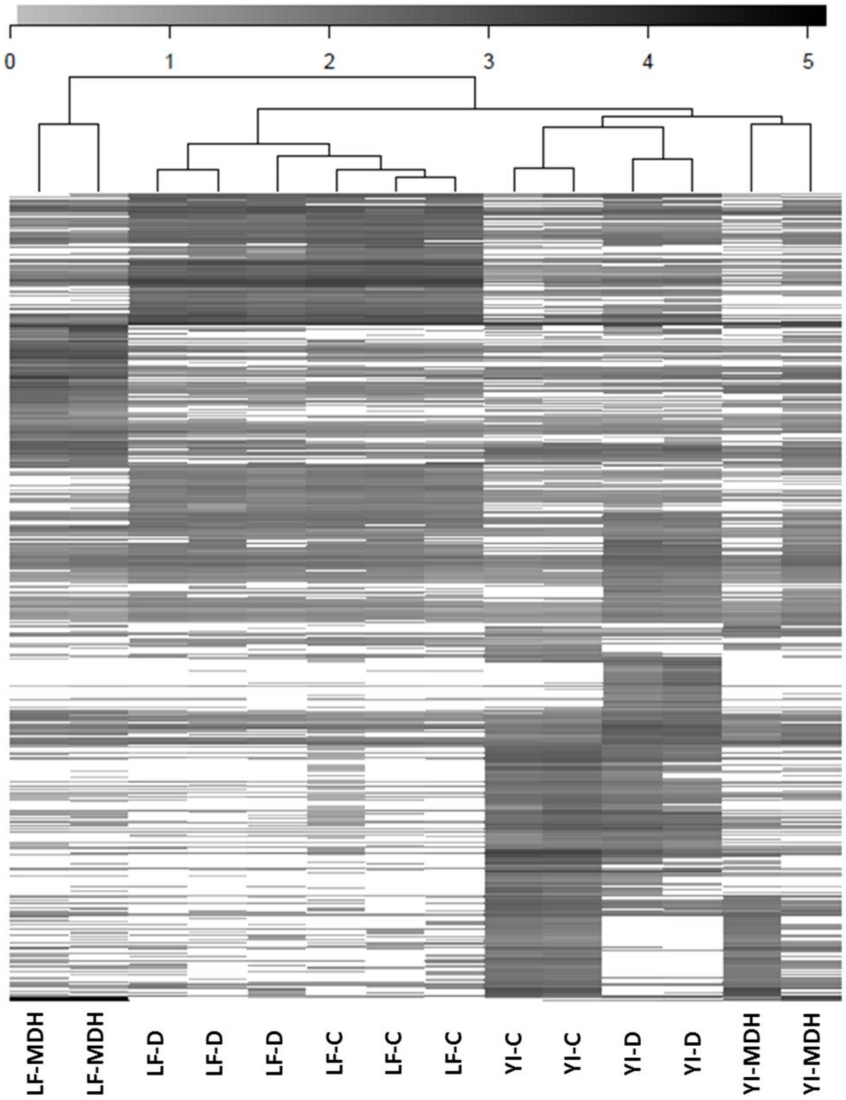


**Figure S11. Heatmap of differentially expressed isoforms after hierarchical clustering.**

Gene expression values are TPM (transcripts per million) from kallisto. Each row shows the relative expression magnitude of an isoform which has been found to be differentially expressed in at least one of the contrasts. Young inflorescences (YI) and leaves (LF); drought, D, and mild drought and heat, MDH.

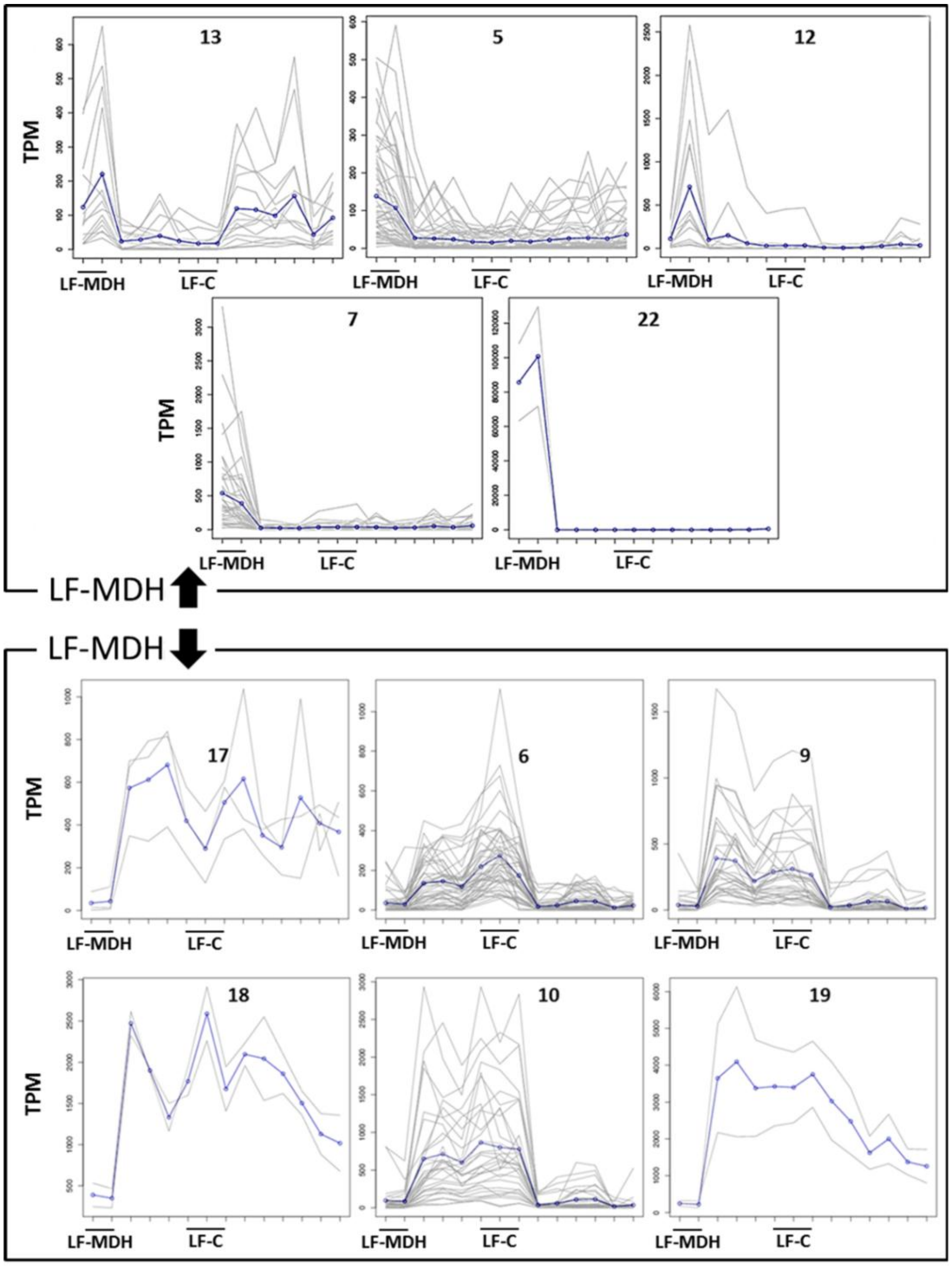


**Figure S12. Clusters with most isoforms declared as differentially expressed in leaf samples in the greenhouse.**

Each plot shows gene expression values across samples, in TPM (transcripts per million), of isoforms in a given cluster (identified by a number within the plot). These plots share a box with other clusters in which most differentially expressed isoforms were found in the same contrast and with the same direction (induced, up arrow; repressed, down arrow). Those samples which are part of the contrast in which the isoforms were differentially expressed are highlighted in the horizontal axis of the plots. LF: leaves, YI: young inflorescences; MDH: mild drought and heat, D: drought, C: control treatments.

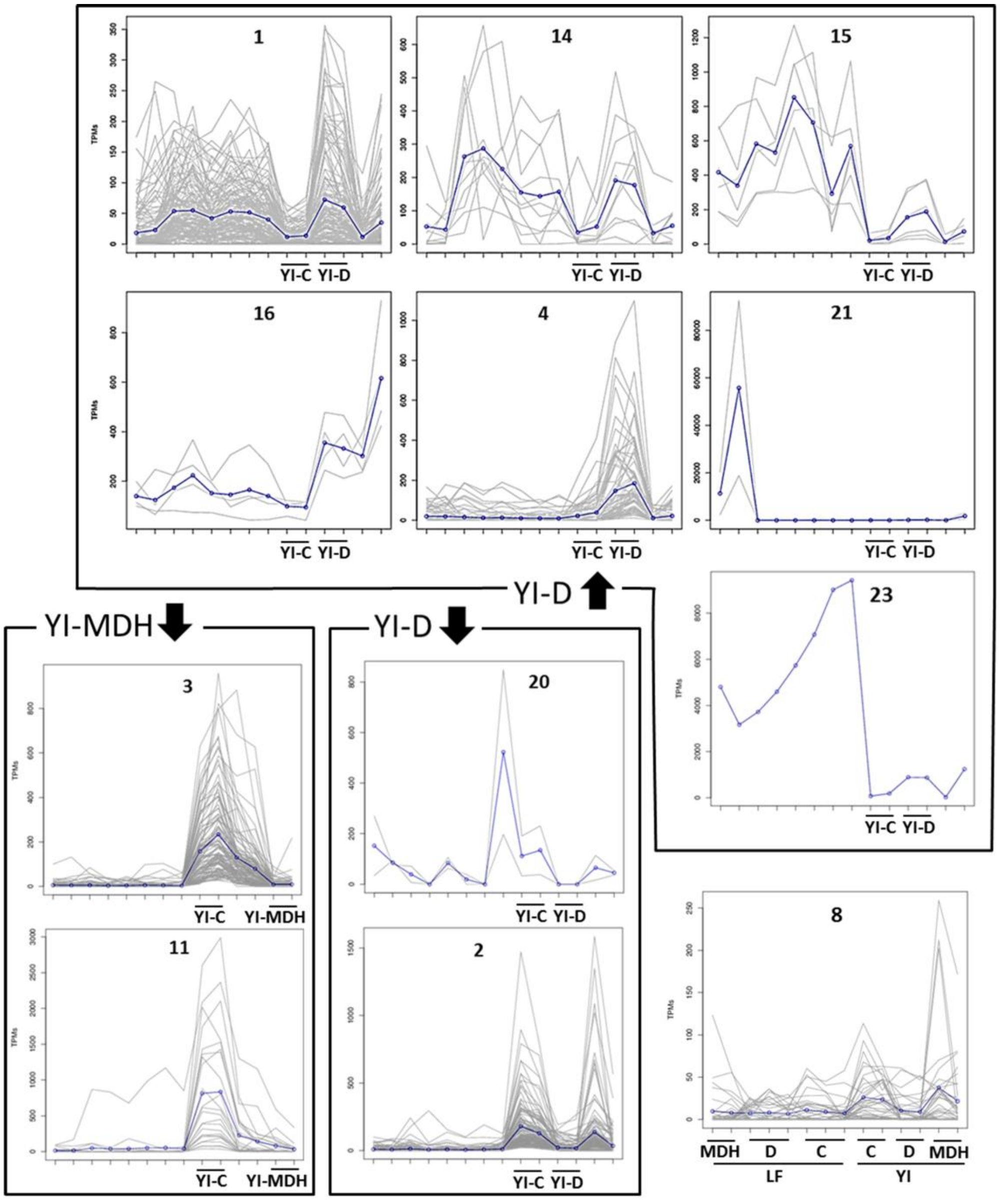


**Figure S13. Clusters with most isoforms declared as differentially expressed in young inflorescences samples.**

Each plot shows gene expression values across samples, in TPM (transcripts per million), of isoforms in a given cluster (identified by a number within the plot). These plots share a box with other clusters in which most differentially expressed isoforms were found in the same contrast and with the same direction (induced, up arrow; repressed, down arrow). Those samples which are part of the contrast in which the isoforms were differentially expressed are highlighted in the horizontal axis of the plots. All samples have been annotated in cluster 8, which has differentially expressed isoforms from different contrasts. LF: leaves, YI: young inflorescences; MDH: mild drought and heat, D: drought, C: control treatments.

## Supplementary Tables

### Table S1. Primers for RT-qPCR validation of RNAseq gene expression values.

Primers are divided in 3 groups: controls chosen from RNAseq data (C1-C3), controls chosen from literature (L1-L3), and RNAseq transcripts whose gene expression could be tested (R1-R12). Sequences are shown both for forward (“F”) and reverse (“R”) primers, including their melting temperature (“Tm”) and amplicon size (“Amp size”). Primers for reference genes were searched in the literature, including the works of Burton et al. 2004 Plant Physiology 134:224-236, Faccioli et al. 2007 Plant Mol Biol 63:679-688, Rapacz et al. 2012 Acta Physiologiae Plantarum 2012 1-11, Janská et al. 2013 Mol Genet and Genomics 288:639-649, Hua et al. 2015 Plant Mol Biol Reporter 1-11 Horvath et al. 2003 (Proc Nat Acad Sci 100:364-369, McGrann et al. Eur J Plant Pathol 123:5-15, Rostoks et al. 2003 107:1094-1101 and Trevaskis et al. 2006 Plant Physiology 140:1397-1405. In total, 14 possible housekeeping genes were located in SBCC073 de novo, Scarlett de novo, Morex WGS and HC/LC Morex assemblies, by alignment with isPCR and re-PCR. Only 2 genes had positive TPM values in all samples from the present work: EIF5A2 from Hua et al and UBI from Rapacz et al, with coefficient of variation (CV) in samples from young inflorescences 0.26 and 0.37, respectively. We also included the widely used primers for Actin from Trevaskis et al, to a final set of 3 reference genes (L1-L3). Further reference genes were selected from our RNAseq data (C1-C3), among those not reported as DE by edgeR. Three isoforms with the lowest coefficient of variation (CV) of TPM values across samples were chosen (CV 0.18-0.25).

| Code | Locus | primer sequence | | Tm | Amp size |
|---|---|---|---|---|---|
| C1 | DCP5 \| decapping 5 | F | AGCAGAGAAAAATAGACACAGAGACATT | 58.3 | 142 |
| | | R | CCTGTATATCCATACCCTCTTCCATAG | 58.4 | |
| C2 | thioredoxin family protein | F | TTTTCTGCAAGGAAGGCAAAC | 58.1 | 87 |
| | | R | GCCCAGCTAACTTTGCAACCT | 59.4 | |
| C3 | SKIP \| chromatin protein family | F | TCTGGTTTTGCTGCTGATGATC | 58.9 | 91 |
| | | R | TCTTGGGCCTGTAAAGACTTGAC | 58.4 | |
| L1 | EIF5A2 from Hua et al 2014 | F | AGGGTGTATGCGGATGTGA | 55.9 | 118 |
| | | R | AATAGCATTCTCGGCTTCCA | 56.2 | |
| L2 | UBI from Rapacz et al 2012 | F | TCGCCGTCCTCCAGTTCTAC | 58.9 | 63 |
| | | R | CCTTCCTGAGCCTGGTTACCT | 58.3 | |
| L3 | ACT from Trevaskis et al 2006 | F | GCTTCTCCTTGATGTCCCTTA | 54.8 | 235 |
| | | R | GCCGTGCTTTCCCTCTATG | 57.0 | |
| R1 | GDSL esterase/lipase | F | GCCGGCAAATACGTCTTCTTC | 59.7 | 68 |
| | | R | GCACCGGCTATCATCCTGTAC | 58.2 | |
| R2 | Germin-like protein 2a | F | GCGCTCTTCGCCAACAA | 58.2 | 63 |
| | | R | CGCGTCGTCCAAGAAGGT | 58.4 | |
| R3 | (unknown) | F | CAAATTGATTCGTTCTAGGTGCAA | 59.2 | 84 |
| | | R | CCGTAGGATGCCAAAGCAA | 58.3 | |
| R4 | Abscisic stress ripening | F | CCGTGAGCCCGTGACAAG | 59.9 | 71 |
| | | R | GCCGTACTGGTCAGCCATCT | 58.8 | |
| R5 | Yellow stripe 1A transporter | F | GCAATTTAGCCACAAGCAGGTT | 59.3 | 74 |
| | | R | GGTGTAACTCCTCAAGCGAGACA | 59.4 | |
| R6 | Lipase | F | GAGGGTCACCCGGAAGACA | 59.5 | 143 |
| | | R | GATGAATTGACGACATGTGCAA | 58.1 | |
| R7 | GDSL esterase/lipase | F | CAGAACTACCTCTCCGGATCCA | 59.2 | 73 |

| | | | | | |
|---|---|---|---|---|---|
| | | R | GGCGGTCGAGGTGTAGGA | 58.2 | |
| R8 | Pre-mRNA-processing-splicing factor | F | ATCGAGTTACCATTCACAAAACCTT | 58.1 | 116 |
| | | R | TGTGTGGATAACCTTCAGAAATAGTTG | 58.6 | |
| R9 | Ubiquitin-conjugating enzyme | F | CATGGGCCCTGGTGATAGC | 59.9 | 117 |
| | | R | GGGTGGTACACCTTTGTTTGGA | 59.6 | |
| R10 | Sugar transporter, putative | F | AGCTAGAGTGCAGAAATTGATTATGC | 58.3 | 79 |
| | | R | CACCAGGGTCAACATCAACTAATTTA | 59.2 | |
| R11 | galacturonosyltransferase-like 4 | F | CGTGCAGAAGCAGAAGAGGAT | 58.3 | 67 |
| | | R | CCCAGCAAGCACTAGAAGGAA | 58.2 | |
| R12 | Pectinesterase | F | GACGAGCCCCACGTGAAG | 58.8 | 117 |
| | | R | TTGCTTGGTGATGCTGTCCTT | 59.3 | |

**Table S2. Intersection of differentially expressed tags between different software pipelines.**

We compared the agreement of the three methods regarding specific genes and isoforms declared as DE (DE tags) by each of them. Intersection statistic ‘s2’ was calculated as the ratio of intersection over union, as described in Boulesteix and Slawski 2009 Briefings in bioinformatics 10:556-568. EdgeR and sleuth had the largest intersection. For those contrasts in which the number of sleuth DE tags was not negligible, those confirmed by edgeR ranged from 51% to 76% for isoforms and from 36% to 81% for genes. The proportion of common DE tags was larger between isoforms (average s2 = 0.1260) than between genes (average s2 = 0.0862). DE isoforms (top) and genes (bottom) found with edgeR, sleuth and cuffdiff are shown (“# DE tags”), for each contrast with genotypes SBCC073 (73) and Scarlett (SC), involving young inflorescences (YI) and leaves (LF) tissues, of plants under drought (D) or mild drought and heat (MDH). The maximum possible intersection between cuffdiff and either edgeR or sleuth corresponds to the total number of DE tags clustered together (“cross linked”). Intersections between each pair of methods and for all of them are shown (“∩”) along with a statistic to relatively score the agreement between them (“s2”).

| **isoforms** | | | | | | | | | | | | | | |
|---|---|---|---|---|---|---|---|---|---|---|---|---|---|---|
| | # DE tags | | | Cross linked | | | edgeR-sleuth | | edgeR-cuffdiff | | sleuth-cuffdiff | | edgeR-sleuth-cuffdiff | |
| Sample | edgeR | sleuth | cuffdiff | edgeR | sleuth | cuffdiff | ∩ | s2 | ∩ | s2 | ∩ | s2 | ∩ | s2 |
| 73-YI-D | 30 | 6 | 36 | 15 | 3 | 26 | 1 | 0.0286 | 0 | 0.0000 | 0 | 0.0000 | 0 | 0.0000 |
| SC-YI-D | 4086 | 513 | 77 | 1976 | 307 | 57 | **334** | **0.0783** | **45** | **0.0226** | **9** | **0.0254** | **8** | **0.0017** |
| 73-YI-M | 110 | 8 | 59 | 52 | 3 | 47 | 5 | 0.0442 | **5** | **0.0532** | 1 | 0.0204 | 0 | 0.0000 |
| SC-YI-M | 912 | 321 | 87 | 456 | 212 | 66 | **165** | **0.1545** | **30** | **0.0610** | **10** | **0.0373** | **9** | **0.0069** |
| 73-LF-D | 435 | 7 | 20 | 225 | 5 | 14 | 4 | 0.0091 | 5 | 0.0214 | 0 | 0.0000 | 0 | 0.0000 |
| SC-LF-D | 256 | 11 | 0 | 121 | 6 | 0 | 5 | 0.0191 | 0 | 0.0000 | 0 | 0.0000 | 0 | 0.0000 |
| 73-LF-MDH | 891 | 149 | 81 | 484 | 81 | 54 | **113** | **0.1219** | **30** | **0.0591** | **4** | **0.0305** | **3** | **0.0027** |
| SC-LF-MDH | 1323 | 386 | 159 | 756 | 251 | 114 | **222** | **0.1493** | **35** | **0.0419** | **37** | **0.1128** | **10** | **0.0054** |

| **genes** | | | | | | | | | | | | | | |
|---|---|---|---|---|---|---|---|---|---|---|---|---|---|---|
| | # DE tags | | | Cross linked | | | edgeR-sleuth | | edgeR-cuffdiff | | sleuth-cuffdiff | | edgeR-sleuth-cuffdiff | |
| Sample | edgeR | sleuth | cuffdiff | edgeR | sleuth | cuffdiff | ∩ | s2 | ∩ | s2 | ∩ | s2 | ∩ | s2 |
| 73-YI-D | 217 | 6 | 51 | 128 | 3 | 40 | 4 | 0.0183 | **11** | **0.0701** | 1 | 0.0238 | 0 | 0.0000 |
| SC-YI-D | 2339 | 434 | 118 | 1405 | 350 | 92 | **158** | **0.0604** | **59** | **0.0410** | **12** | **0.0279** | **9** | **0.0031** |
| 73-YI-MDH | 122 | 5 | 72 | 72 | 3 | 57 | 5 | 0.0410 | **6** | **0.0488** | 1 | 0.0169 | 0 | 0.0000 |
| SC-YI-MDH | 2081 | 292 | 156 | 1351 | 231 | 126 | **217** | **0.1006** | **78** | **0.0558** | **22** | **0.0657** | **15** | **0.0060** |
| 73-LF-D | 887 | 7 | 48 | 678 | 7 | 38 | 6 | 0.0068 | **15** | **0.0214** | 0 | 0.0000 | 0 | 0.0000 |
| SC-LF-D | 180 | 11 | 17 | 139 | 8 | 13 | 6 | 0.0324 | 6 | 0.0411 | 0 | 0.0000 | 0 | 0.0000 |
| 73-LF-MDH | 1589 | 139 | 207 | 1174 | 115 | 170 | **113** | **0.0700** | **99** | **0.0795** | **21** | **0.0795** | **17** | **0.0089** |
| SC-LF-MDH | 1719 | 309 | 287 | 1312 | 246 | 221 | **207** | **0.1137** | **108** | **0.0758** | **48** | **0.1146** | **26** | **0.0114** |

**Table S3. Intersection between differentially expressed isoforms and genes.**

Given that each isoform is associated to a gene, we compared the results from the differential expression test carried out either based on isoforms or directly based on genes. The total number of DE genes detected by both approaches ("common tags") is shown for each contrast with genotypes SBCC073 (73) and Scarlett (SC), involving young inflorescences (YI) or leaves (LF), of plants subjected to drought (D) or to mild drought and heat (MDH). Intersection statistics include the percentage of common DE genes in relation to the maximum possible intersection ("%") and the "s2" statistic (see Methods).

| contrast | isoforms | genes | common tags | % | s2 |
|---|---|---|---|---|---|
| 73-YI-D | 1 | 4 | 1 | 100% | 0.2500 |
| SC-YI-D | 334 | 158 | 110 | 70% | 0.2880 |
| 73-YI-MDH | 5 | 5 | 5 | 100% | 1.0000 |
| SC-YI-MDH | 165 | 217 | 150 | 91% | 0.6466 |
| 73-LF-D | 4 | 6 | 4 | 100% | 0.6667 |
| SC-LF-D | 5 | 6 | 4 | 80% | 0.5714 |
| 73-LF-MDH | 113 | 113 | 102 | 90% | 0.8226 |
| SC-LF-MDH | 222 | 207 | 186 | 90% | 0.7654 |

**Table S4. Correlation between differential expression analysis and physiological measurements.**

Pearson correlation of physiological measurements with logFC and number of DE (differentially expressed) tags (left subtable), for genes (top) and isoforms (bottom), both in young inflorescences (YI) and leaves (LF). P-values for each correlation coefficient (right subtable). LWP: leaf water potential. SCo: stomatal conductance. RWC: relative water content. TN: tiller number. VSN: visible spike number.

| | | | Pearson r | | | | | p-values | | | | |
|---|---|---|---|---|---|---|---|---|---|---|---|---|
| | | | LWP | SCo | RWC | TN | VSN | LWP | SCo | RWC | TN | VSN |
| genes | YI | logFC | 0.5050 | 0.6634 | -0.1720 | -0.8264 | -0.4042 | 0.4950 | 0.3366 | 0.8280 | 0.1736 | 0.5958 |
| | | DE tags | 0.6907 | 0.4739 | -0.1728 | -0.7311 | -0.2005 | 0.3093 | 0.5261 | 0.8272 | 0.2689 | 0.7995 |
| | LF | logFC | -0.1641 | 0.9801 | 0.1084 | -0.7106 | -0.9074 | 0.8359 | 0.0199 | 0.8916 | 0.2894 | 0.0926 |
| | | DE tags | -0.2637 | 0.9532 | 0.2866 | -0.5567 | -0.9596 | 0.7363 | 0.0468 | 0.7134 | 0.4433 | 0.0404 |
| isofs | YI | logFC | 0.9701 | -0.0730 | -0.0807 | -0.3162 | 0.3023 | 0.0299 | 0.9270 | 0.9193 | 0.6838 | 0.6977 |
| | | DE tags | 0.9881 | -0.1703 | -0.0672 | -0.2330 | 0.3855 | 0.0119 | 0.8297 | 0.9328 | 0.7670 | 0.6145 |
| | LF | logFC | -0.1980 | 0.9745 | 0.1738 | -0.6586 | -0.9297 | 0.8020 | 0.0255 | 0.8262 | 0.3414 | 0.0703 |
| | | DE tags | -0.2433 | 0.9595 | 0.2592 | -0.5839 | -0.9523 | 0.7567 | 0.0405 | 0.7408 | 0.4161 | 0.0477 |

**Table S5. Protein domains annotated within peptides encoded by differentially expressed isoforms in leaves under mild drought and heat.**

Differentially expressed isoforms not directly associated to metabolic pathways or specific cellular processes are listed, such as transcription factors and regulators, protein kinases, transporters and other protein domains. Presence among DE isoforms in SBCC073 (73) and Scarlett (SC) is marked with background color. Green: up-regulated ("Up"). Red: down-regulated.

| **Transport** | 73 | SC |
|---|---|---|
| SPX domain-containing Major Facilitator protein | Up | |
| Anion transporter (HCO3-) | Up | |
| Putative phosphate transporter | | Up |
| ZIP3 Zn Transporter | | |
| Potassium channel AKT2 | | Up |
| TIP4-1 aquaporin | | |
| PIP2-3 aquaporin | | |
| Triose-phosphate transporter | | Up |
| Monosaccharide-sensing protein2 | | |
| Probable mitoch. adenine nucl. transporter | | |
| Probable lipid transfer | | Up |
| Plant lipid transfer protein (LTP) | | Up |
| Vacuolar amino acid transporter | | Up |
| MatE | Up | |
| ABC transporter C-14 | | Up |
| ABC transporter G-11 | | Up |
| Exo70 exocyst complex subunit | | |
| protein NUCLEAR FUSION DEFECTIVE 4 | | |
| **Protein kinases and phosphatases** | | |
| CIPK9 | Up | Up |
| CIPK17 | Up | |
| RLK-Pelle_LRR-XII-1 | Up | |
| AGC_RSK-2 | | |
| Calcineurin-like phosphoesterase | | Up |
| Inorganic Ppase | Up | |
| **Transcription factors** | | |
| Myb-related TF CCA1/LHY | Up | Up |
| Myb-related TF | Up | Up |

| **Other domains** | 73 | SC |
|---|---|---|
| Alpha-beta hydrolase | Up | Up |
| RNA helicase DEAD-box | | |
| Pumilio-homolog 7, chloroplastic | | |
| Mitochondrial RNA recognition motif | | |
| ROOT PRIMORDIUM DEFECTIVE 1 | | |
| ATP synthase protein I | | |
| hAT family C-terminal dimerisation region | | |
| Sigma-70 TI | | |
| RPL27 | | |
| Small ribosomal protein S8 | | Up |
| Small ribosomal RNA 18 | | Up |
| Myosin V | Up | |
| Actin-depolymerizing factor 7 (cofilin) | | |
| Jacalin-like lectin / Dirigent-like protein | | |
| Rhodanase-like / HpcH/HpaI aldolase | | |
| UV-B-induced protein, chloroplastic | Up | |
| SPFH domain / Band 7 | Up | |
| F-box protein SKIP14 | | Up |
| Lethal giant larvae like | | Up |
| WD40-like Beta Propeller Repeat | | Up |
| UMP kinase | | |
| Tetratricopeptide repeat | | |
| CRAL/TRIO domain | | |
| VMA21-like domain | | |
| D-mannose biding lectin | | |
| **Signal transduction, protein interactions** | | |
| Adagio-like protein 3 | | |
| PRR1 TR | | |

| | | |
|---|---|---|
| C2C2-Dof TF | Up | |
| AP2/ERF-AP2 | | |
| TUB | | |
| bZIP | | Up |
| Tify | | Up |

| | | |
|---|---|---|
| APRR3 TR | | |
| MBF1 TR | | |
| NPY3 (NPH3 family): positive gravitropism | | |
| ASPR2 topless-related protein 2 | Up | |
| Pseudo ARR-B TR | | Up |
| Uncharacterized iTAK TR | | |
| Zn finger CONSTANS-like 9 | | |

upregulated Up

downregulated

**Table S6. Protein domains annotated within peptides encoded by differentially expressed isoforms in Scarlett young inflorescences.**

Differentially expressed isoforms not directly associated to metabolic pathways or specific cellular processes are listed, such as transcription factors and regulators, protein kinases, transporters and other domains. Presence among DE isoforms in the growth chamber (D) and the greenhouse (MDH) is marked with background color. Green: upregulated (“Up”). Red: downregulated.

| Transport | D | MDH |
|---|---|---|
| Bidirectional sugar transporter SWEET | | |
| Sugar (and other) transporter | | |
| Triose-phosphate transporter | Up | |
| Lysine histidine transporter | | |
| PRA1 family protein B2 | | |
| ABC transporter B1 | | |
| ABC transporter G51 | | |
| ABC transporter C14 | | |
| ABC-2 type transporter | | |
| ABC transporter E1 | Up | |
| PIP2-6 aquaporin | | |
| NIP1-4 aquaporin | | |
| Ca2+:H+ antiporter | | |
| Calcium-transporting ATPase | Up | |
| Potassium transporter | Up | |
| Boron transporter 1 | Up | |
| Clathrin heavy chain | Up | |
| Coatomer subunit gamma | | |
| Dynamin-related protein 5A | | |
| Dynamin GTPase | | |
| SRP receptor subunit alpha | | |
| epsin | | |
| Importin-bea, exportin 1-like, Cse1 | Up | |
| Nuclear cap-binding protein subunit 1 | Up | |
| Nuclear-pore anchor TRP/MLP1/MLP2-like | Up | |
| **Protein kinases and phosphatases** | | |
| CMGC_GSK | | |
| CMGC_MAPK | | |

| RNA processing, translation | D | MDH |
|---|---|---|
| IF-1 | Up | |
| IF-4G | Up | |
| IF-3A | Up | |
| eIF2A | | |
| eIF2B/IF5 | | |
| EF-1 | | Up |
| EF-1beta | Up | |
| EF-1alpha | Up | |
| EF G, chloroplastic | Up | |
| Valyl-tRNA synthetase | Up | |
| Glycyl-tRNA synthetase | | |
| Ribosomal protein L30p/L7e | | |
| Large subunit ribosomal L7Ae/L30e/S12e/Gadd45 | | |
| Piwi-AGO protein | Up | |
| SUPPRESSOR OF GENE SILENCING 3 homolog | | |
| Small subunit processome component 20 homolog | Up | |
| DNA-directed RNA polymerase V subunit 1 | Up | |
| Poly(U)-specific endoribonuclease | Up | |
| EF of RNApol II, NGN section | Up | |
| Pumilio family | Up | |
| 16S rRNA methyltransferase | Up | |
| **Cytoskeleton** | | |
| Tubulin C-terminal domain | | |
| Myosin-17 | | |
| Fimbrin-5 | Up | |
| Tubulin alpha | Up | |
| Villin-3 | Up | |
| Calponin homology domain | Up | |

| | | |
|---|---|---|
| OSK1 5'-AMP-activated PK | | |
| Cyclin-dependent kinase 7 | | |
| CIPK30 | Up | |
| calcium-dependent protein kinase 3 | Up | |
| calcium-dependent protein kinase 7 | Up | |
| casein kinase I isoform delta-B | Up | |
| Ser/Thr-protein kinase mTOR | Up | |
| UMP-CMP kinase | | |
| Phosphoinositide phosphatase SAC7 | Up | |
| **Signal transduction, protein interactions** | | |
| SNF2 TR | Up | |
| ASPR1 topless-related protein 1 | Up | |
| 14-3-3 protein epsilon | Up | |
| 14-3-3 protein epsilon | | |
| brefeldin A-inhibited GNP-exchange protein | Up | |
| brefeldin A-inhibited GNP-exchange protein | | |
| CypP450 | Up | |
| PB1 Zn finger Ig-like | Up | |
| Rubisco activase, chloroplastic | Up | |
| Phospholipase A-2-activating protein | | |
| **Transcription factors** | | |
| B3-ARF | Up | Up |
| MADS-MIKC | Up | |
| Myb-related TF CCA1/LHY | | Up |
| **Cell wall** | | |
| Pectinesterase | | |
| Pectinesterase | Up | |
| Expansin-A7 | | |
| Expansin-B3 | | |
| Putative cell wall protein | | |
| PMR5/Cas1p | | |

| | | |
|---|---|---|
| Kinesin motor domain | Up | |
| Kinesin-like protein C2/C3 | | Up |
| Actin depolymerizing factor 6 | | |
| **Other domains** | | |
| ATP-dependent RNA helicase DDX3X | Up | |
| GUCT (NUC152) domain | | |
| U3 small nucleolar RNA-associated protein 10 | Up | |
| Methyl-CpG binding domain | Up | |
| Tudor domain | Up | |
| GTP-binding protein 2 | Up | |
| Vps4 C-terminal oligomerisation domain | | |
| DNA topoisomerase 2 | | Up |
| Germin-like protein 5-1 | | |
| Putative germin-like protein 3-2 | | |
| 12S seed storage protein CRB | | |
| Sulfotransferase | | |
| Thiosulfate sulfurtransferase | Up | |
| Cytochrome b561 | | |
| Pollen proteins Ole e I like | | |
| Plastocyanin-like domain | | |
| Endomembrane protein 70 | | |
| Filament-like plant protein, long coiled-coil | | |
| UvrD/REP helicase | | |
| Fascilin-like arabinogalactan protein 11 | | |
| Condensin complex subunit 1 | | Up |
| alpha-L-fucosidase 2 | Up | |
| alpha-L-fucosidase 2 | | |
| protein TIF31 | | |

upregulated Up
downregulated

**Table S7. Size and composition of each cluster of co-expressed differentially expressed isoforms.**

Each row corresponds to a cluster with a numeric identifier (“ID”) and its size (number of co-expressed differentially expressed isoforms). Each of those isoforms correspond to one or more contrasts (genotypes SBCC073, “73”, and Scarlett, “SC”; young inflorescences, “YI”, and leaves, “LF”, tissues; drought, “D”, and mild drought and heat, “MDH”, experiments). Red background gets darker as the contribution of each cluster to each contrast increases.

| | | LF-D | | | | LF-MDH | | | | YI-D | | | | YI-MDH | | | |
|---|---|---|---|---|---|---|---|---|---|---|---|---|---|---|---|---|---|
| | | 73 | | SC | | 73 | | SC | | 73 | | SC | | 73 | | SC | |
| ID | size | up | dn | up | dn | up | dn | up | dn | up | dn | up | dn | up | dn | up | dn |
| 8 | 34 | 0 | 0 | 0 | 1 | 0 | 2 | 6 | 6 | 0 | 0 | 0 | 8 | 0 | 0 | 6 | 5 |
| 20 | 2 | 0 | 0 | 0 | 0 | 0 | 0 | 0 | 0 | 0 | 0 | 0 | 2 | 0 | 0 | 0 | 0 |
| 2 | 130 | 0 | 0 | 0 | 0 | 0 | 1 | 5 | 0 | 0 | 1 | 0 | 103 | 0 | 0 | 0 | 22 |
| 11 | 23 | 0 | 0 | 0 | 0 | 0 | 0 | 0 | 1 | 0 | 0 | 0 | 8 | 0 | 0 | 0 | 15 |
| 3 | 109 | 0 | 0 | 0 | 0 | 0 | 1 | 1 | 1 | 0 | 0 | 0 | 2 | 0 | 1 | 0 | 104 |
| 13 | 15 | 0 | 0 | 0 | 0 | 0 | 0 | 15 | 0 | 0 | 0 | 0 | 0 | 0 | 0 | 0 | 0 |
| 12 | 16 | 2 | 0 | 0 | 0 | 4 | 0 | 10 | 0 | 0 | 0 | 0 | 0 | 0 | 0 | 0 | 0 |
| 5 | 63 | 0 | 0 | 1 | 0 | 17 | 0 | 41 | 0 | 0 | 0 | 1 | 1 | 3 | 0 | 1 | 0 |
| 7 | 47 | 0 | 0 | 0 | 0 | 28 | 0 | 19 | 0 | 0 | 0 | 0 | 0 | 1 | 0 | 0 | 0 |
| 22 | 2 | 0 | 0 | 0 | 0 | 0 | 0 | 2 | 0 | 0 | 0 | 0 | 0 | 0 | 0 | 0 | 0 |
| 18 | 2 | 0 | 0 | 0 | 0 | 0 | 2 | 0 | 0 | 0 | 0 | 0 | 0 | 0 | 0 | 0 | 0 |
| 9 | 33 | 0 | 0 | 0 | 0 | 0 | 24 | 0 | 7 | 0 | 0 | 2 | 0 | 0 | 0 | 0 | 0 |
| 10 | 31 | 0 | 0 | 0 | 0 | 0 | 4 | 0 | 25 | 0 | 0 | 2 | 0 | 0 | 0 | 0 | 1 |
| 17 | 3 | 0 | 0 | 0 | 0 | 0 | 1 | 0 | 2 | 0 | 0 | 0 | 0 | 0 | 0 | 0 | 0 |
| 19 | 2 | 0 | 0 | 0 | 0 | 0 | 0 | 0 | 2 | 0 | 0 | 0 | 0 | 0 | 0 | 0 | 0 |
| 6 | 47 | 0 | 0 | 0 | 2 | 0 | 8 | 0 | 27 | 0 | 0 | 10 | 0 | 0 | 0 | 0 | 1 |
| 1 | 166 | 0 | 0 | 0 | 1 | 0 | 14 | 5 | 37 | 0 | 0 | 108 | 1 | 0 | 0 | 0 | 0 |
| 14 | 11 | 2 | 0 | 0 | 0 | 0 | 4 | 0 | 0 | 0 | 0 | 5 | 0 | 0 | 0 | 0 | 0 |
| 15 | 6 | 0 | 0 | 0 | 0 | 0 | 0 | 0 | 0 | 0 | 0 | 6 | 0 | 0 | 0 | 0 | 0 |
| 16 | 4 | 0 | 0 | 0 | 0 | 0 | 0 | 0 | 0 | 0 | 0 | 4 | 0 | 0 | 0 | 1 | 0 |
| 4 | 86 | 0 | 0 | 0 | 0 | 3 | 0 | 9 | 0 | 0 | 0 | 68 | 0 | 0 | 0 | 0 | 9 |
| 21 | 2 | 0 | 0 | 0 | 0 | 0 | 0 | 1 | 0 | 0 | 0 | 2 | 0 | 0 | 0 | 0 | 0 |
| 23 | 1 | 0 | 0 | 01 | 0 | 0 | 0 | 0 | 0 | 0 | 0 | 1 | 0 | 0 | 0 | 0 | 0 |
